# The first megatheropod tracks from the Lower Jurassic upper Elliot Formation, Karoo Basin, Lesotho

**DOI:** 10.1371/journal.pone.0185941

**Published:** 2017-10-25

**Authors:** L. Sciscio, E. M. Bordy, M. Abrahams, F. Knoll, B. W. McPhee

**Affiliations:** 1 Department of Geological Sciences, University of Cape Town, Cape Town, South Africa; 2 Fundación Conjunto Paleontológico de Teruel-Dinópolis, Teruel, Spain; 3 Departamento de Biologia, FFCLRP, Universidade de São Paulo, São Paulo, Brazil; Institute of Botany, CHINA

## Abstract

A palaeosurface with one megatheropod trackway and several theropod tracks and trackways from the Lower Jurassic upper Elliot Formation (Stormberg Group, Karoo Supergroup) in western Lesotho is described. The majority of the theropod tracks are referable to either *Eubrontes* or *Kayentapus* based on their morphological characteristics. The larger megatheropod tracks are 57 cm long and have no Southern Hemisphere equivalent. Morphologically, they are more similar to the Early Jurassic *Kayentapus*, as well as the much younger Upper Cretaceous ichnogenus *Irenesauripus*, than to other contemporaneous ichnogenera in southern Africa. Herein they have been placed within the ichnogenus *Kayentapus* and described as a new ichnospecies (*Kayentapus ambrokholohali*). The tracks are preserved on ripple marked, very fine-grained sandstone of the Lower Jurassic upper Elliot Formation, and thus were made after the end-Triassic mass extinction event (ETE). This new megatheropod trackway site marks the first occurrence of very large carnivorous dinosaurs (estimated body length >8–9 meters) in the Early Jurassic of southern Gondwana, an evolutionary strategy that was repeatedly pursued and amplified in the following ~135 million years, until the next major biotic crisis at the end-Cretaceous.

## Introduction

During the first 30 million years of their evolution, dinosaurs constituted a relatively morphologically non-diverse group of land vertebrates compared to contemporaneous crurotarsans [1, 2] with which they shared many Late Triassic ecosystems. The outset of the Jurassic witnessed the global evolutionary radiation of Dinosauria, with events associated with the end-Triassic mass extinction (ETE) and the Triassic-Jurassic boundary (TJB) often hypothesised to have played an important role [3]. The earliest Jurassic is thus a period of particular interest as it spans a post-extinction recovery period during which dinosaurs continued to thrive and diversify globally [3, 4]. This observation has been instrumental in recent interpretations that favour abiotic contingency (“opportunism”) [5, 6] over competitive superiority in explaining the ultimate success of the dinosaurs [3, 7].

During the Late Triassic and Early Jurassic, the largest carnivorous dinosaurs seldom surpassed 5 m in body length, as evidenced by both the skeletal and ichnofossil record [5]. Olsen et al. [5] suggested that “ecological release” associated with the disappearance of incumbent non-dinosaurian archosaurian predators across the TJB possibly explains the sudden leap in carnivorous dinosaur size thresholds in the Early Jurassic, as evidenced by the ichnospecies *Eubrontes giganteus*.

Thus, body size constraints have been linked to shifts in global ecosystem composition, with medium to larger sized dinosaurs argued to be able to command a greater range of morphospace following the ETE [2, 3]. This hypothesis, which potentially explains the appearance of relatively large-bodied theropod dinosaurs within the earliest Jurassic, was based mainly on the ichnology of the Newark Supergroup (USA) and has been received with some scepticism e.g., [8, 9].

Amongst dinosaurs, Theropoda is remarkable for demonstrating continuous evolutionary novelty throughout the history of the clade, a phenomenon which is particularly noticeable at nodes proximate to the theropod-bird transition [10]. In the last decade, there have been many studies interested in dinosaurian, and specifically theropod, macroevolutionary patterns, with the dynamics of changing body size playing a central role within this literature [2, 3, 10]. However, reconstructing early theropod evolutionary patterns is much more problematic, with studies focused on discrete (morphological) characters depicting gradualistic rates of change for the group across the TJB [2]. In contrast, investigations utilising mass-estimates suggest a significant increase in body size for Theropoda within the Late Triassic [10]. A source of this confusion is the lack of theropod body fossils from this period. Theropod body fossils from the Early Jurassic are relatively rare, with *Coelophysis* bearing the largest number of well-preserved specimens globally [11, 12].

In contrast, the theropod ichnite record is comparatively rich and represents a good source of additional information independent of skeletal material. In general, Upper Triassic strata are dominated by an abundance of theropod ichnites between 20 cm to 25 cm in length (grallatorid-sized) [8]. However, there does appear to be a preponderance for an increase in their size in the Early Jurassic based on the increased number of larger theropod ichnites, with the oldest known example of *Eubrontes* (35 cm) in North America (in a unit that is 10 ky younger than TJB-defined by palynomorphs [5]) and 34 cm in South Africa [13]. Despite the abundance of these Lower Jurassic theropod tracks and trackways, they are currently of relatively low generic diversity due to similarities in track morphology. This limits descriptions to within the one plexus of three ichnogenera (i.e. *Grallator*, *Anchisauripus* and *Eubrontes*) which are primarily distinguished by differences in size [8, 14].

**Fig 1:**
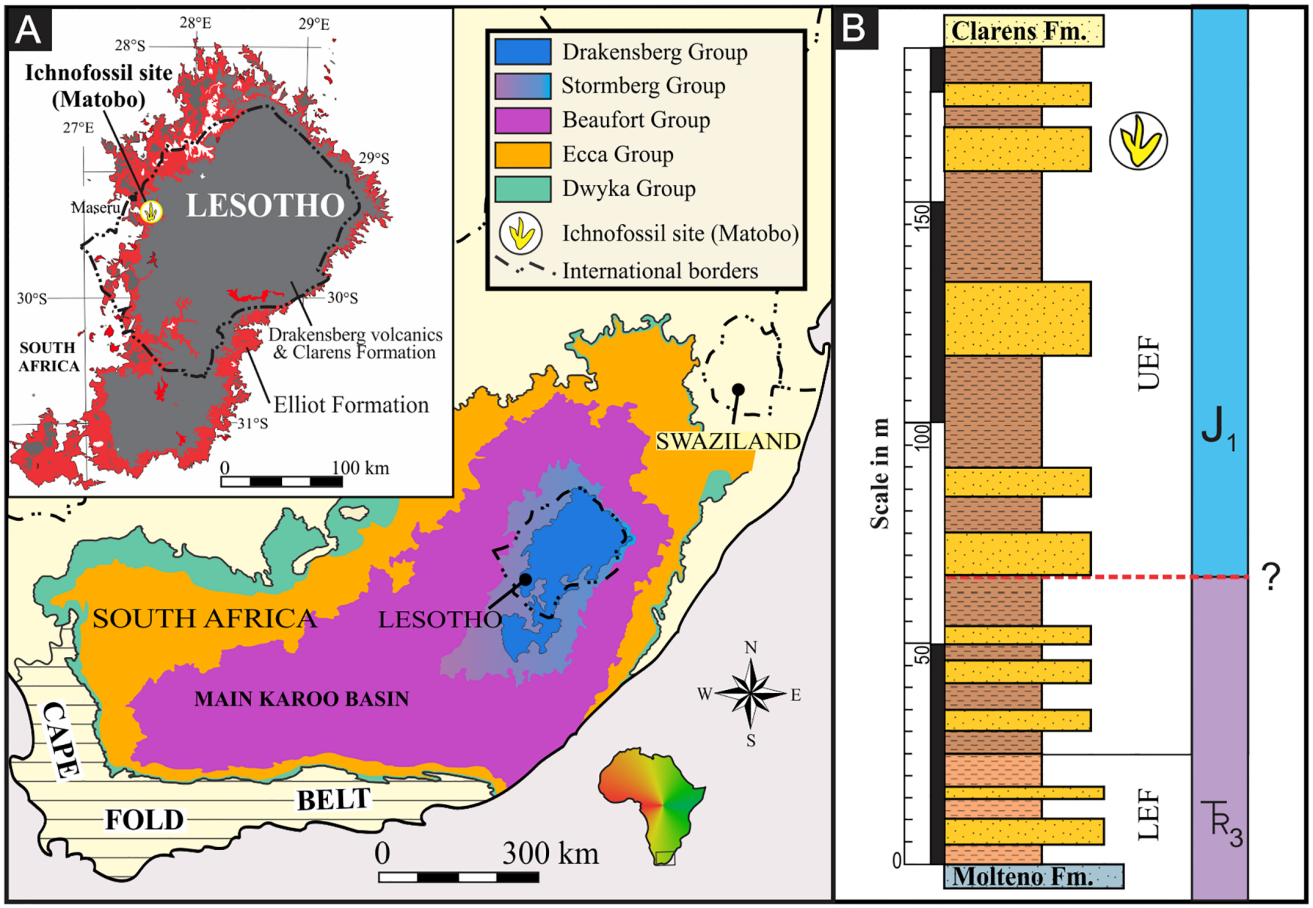
Geological context of the megatheropod trackway (Matobo) site in the Roma Valley (Maseru District, Lesotho). (A) Location of the study area in western-central Lesotho within the main Karoo Basin of southern Africa. The inset shows the location of the site (footprint logo) and spatial distribution of the Upper Triassic to Lower Jurassic Elliot Formation. (B) Generalized sedimentary log of the Elliot Formation at the megatheropod trackway site. Stratigraphic position of the megatheropod trackway site is shown with the footprint logo. LEF—lower Elliot Formation; UEF—upper Elliot Formation. Geological map has been modified from the Simplified Geology map (1:1000 000) of South Africa, Lesotho and Swaziland under a CC BY license, with permission from the CGS, original copyright [2003].

In southern Africa, rare theropod body fossils and isolated teeth are present in Lower Jurassic rocks [15–18]; whereas theropod tracks are common from the Late Triassic (e.g. [19]) and into the Early Jurassic (e.g. [20, 21]). These tridactyl tracks and trackways, despite their conservative foot morphology, allow for a more holistic treatment of theropod evolution, over the TJB. Here, we report, for the first time, a tridactyl dinosaur trackway site from the Elliot Formation in the Roma Valley (Maseru District, Lesotho; Fig 1) that preserves the largest Early Jurassic theropod trackway to date.

This new ichnofossil data from western Lesotho considerably expands the range of body-size displayed by carnivorous dinosaurs in the Early Jurassic in Gondwana, providing insight into the rate and tempo of body-size increase experienced by Theropoda across the ETE and the TJB (i.e., a punctuated, “release-type” versus a more step-wise body-size increase mirroring that of large-bodied dinosaurian herbivores, represented primarily by Sauropodomorpha). In addition to the morphological description of the tracks, we also document the associated taphono-sedimentary context of the trackway site and, using photogrammetry, provide a three-dimensional view to illustrate the morphology of the tracks. Large tracks, herein, refer to any ichnite greater than 40 cm in length and megatheropod tracks refer to the tridactyl theropod tracks >50 cm.

### Geological background and stratigraphy of the megatheropod trackway site

The Matobo megatheropod trackway site is located within the uppermost Elliot Formation, 1.8 km west of the National University of Lesotho main entrance in the Roma Valley (Maseru District, Lesotho; Fig 1). It lies on an informal road between the villages of Ha Mokhosi and Ha Matobo. Although the megatheropod trackways were discovered by the authors, the site is immediately adjacent to the Matobo trackway site that was briefly documented by Ambrose [22].

The Roma Valley itself is carved into the Lower Jurassic successions of the upper Stromberg and lower Drakensberg Groups of the Karoo Supergroup (Fig 1B). The valley floor and sides expose the sedimentary rocks of the upper Elliot and Clarens formations, whereas the hilltops are often capped by Karoo continental flood basalts that were dated at 183±1.0 Ma [23]. Outcrops of the older, Triassic-age rocks (e.g., Molteno and lower Elliot formations) are scarce and limited to the westernmost part of the valley, while mafic dolerite intrusions (also part of the Drakensberg Group) are relatively common.

Stratigraphically, the megatheropod trackway site is found within the Lower Jurassic upper Elliot Formation (Fig 1B and 1C), which is well-documented because of its diverse and abundant vertebrate track assemblages (e.g., [13, 20, 21, 24–28]). Detailed palaeontological, stratigraphic and sedimentological accounts of the Upper Triassic-Lower Jurassic fluvio-lacustrine Elliot and Clarens formations of southern Africa are presented, among others, in [29–40].

The Roma Valley has yielded only a modest assemblage of vertebrate fossils [22]; however, the area has a rich ichnological record. For example, Ambrose [22] described, albeit very briefly, 14 fossil vertebrate track sites located in or close to the Roma Valley and incorporated some drawings by the members of the 1998 British Schools Exploring Society Expedition. Despite intensive searches, our team could only relocate some of the sites listed in Ambrose [22]. This is most probably because the sites were destroyed by local building-stone quarrying activities, which are currently under way informally.

## Material and methods

Field work was conducted under a field permit (permit number: NR/M/E/10) issued by the Lesotho Government Department of Mines and Geology. Field evidence was collected in the form of macroscopic observations of the ichnofossil bearing sedimentary rocks and their vertical and lateral distribution at the study locality, Matobo site (29° 27′ 08.57″S, 27° 42′ 08.51″E; Roma, Maseru District). The outcrop was photographed and described with enough detail to produce an in-depth characterization of the sedimentary facies, which entailed the documenting of lithological, geometric, and sedimentary structures etc. The dinosaur tracks were measured *in situ* and recorded in detail via photographs, photomosaics, and sketches using ImageJ software. Matobo trackways and tracks have been labelled alphabetically ‘A’–‘D’. Where applicable, track length (TL), track width (TW), length of digits (te: II, III, IV), interdigital angles (II^III, III^IV, II^IV), metatarsophalangeal length (FL-te), pace length (PL), stride length (SL), and pace angulation (PANG) were measured for each trackway (Table 1). Track length (TL) is considered, here, as the length from the tip (but not including claw mark where preserved) of digit III to the base of the heel margin. Track measurements were taken as described in [13].

**Table 1:** Measurements of the tracks of trackways A to D at Matobo.

Matobo track	TL	TW	TL/TW	MPL (TL-te)	Digit III extension (te)	(TL-te)/ TW	te/TW	Interdigit angle			Surface Area					Ratios	
								II^IV (°)	II^III (°)	III^IV (°)	MSA	Digit II	Digit III	Digit IV	Total digit SA	MSA/total digit SA	MPL/total length
A1	36	25.5	1.4	24	12.5	0.9	0.5	53	27	26	140	51	71	38	159	0.9	0.7
A2	31	-	-	-	-	-	-	-	-	-	-	-	-	-	-	-	-
A3	30	25	1.2	21	9	0.8	0.4	62	32	30	115	64	80	46	190	0.6	0.7
A4	31	21	1.5	21	10	1.0	0.5	52	24	28	155	45	107	55	208	0.7	0.7
A5	31	22	1.4	20	11	0.9	0.5	58	29	29	112	32	76	73	180	0.6	0.6
*Average*	*32*	*23*	*1*.*4*	*21*.*4*	*10*.*6*	*0*.*9*	*0*.*5*	*56*	*28*	*28*	*130*	*48*	*84*	*53*	*184*	*0*.*7*	*35*.*6*
*S*.*D*.	*2*.*1*	*1*.*9*	*0*.*1*	*1*.*3*	*1*.*3*	*0*.*1*	*0*.*1*	*4*	*3*	*2*	*18*	*12*	*14*	*13*	*18*	*0*.*1*	*5*.*6*
B1	41	31	1.3	29	12	1.0	0.4	57	27	29	406	42	117	31	189	2.1	0.7
B2	39	34	1.1	24	15	0.7	0.4	57	26	30	309	115	201	88	404	0.8	0.6
*Average*	*40*	*32*	*1*.*2*	*26*.*5*	*13*.*5*	*0*.*8*	*0*.*4*	*56*.*9*	*26*.*5*	*29*.*3*	*357*	*79*	*159*	*59*	*297*	*1*.*5*	*145*.*5*
*S*.*D*.	*1*.*4*	*2*.*5*	*0*.*1*	*3*.*5*	*2*.*1*	*0*.*2*	*0*.*0*	*0*.*1*	*0*.*4*	*1*.*1*	*69*	*52*	*60*	*40*	*152*	*1*.*0*	*97*.*7*
C1	30	21	1.5	20	10	1.0	0.5	58	28	30	174	66	149	107	322	0.5	0.7
C2	32	22	1.5	18	14	0.8	0.7	59	31	28	94	48	54	31	133	0.7	0.6
*Average*	*31*	*21*	*1*.*5*	*19*.*0*	*12*.*0*	*0*.*9*	*0*.*6*	*58*.*4*	*29*.*7*	*28*.*7*	*134*	*57*	*102*	*69*	*228*	*0*.*6*	*62*.*2*
*S*.*D*.	*1*.*4*	*0*.*7*	*0*.*0*	*1*.*4*	*2*.*8*	*0*.*1*	*0*.*1*	*0*.*8*	*1*.*9*	*1*.*1*	*57*	*13*	*67*	*54*	*134*	*0*.*1*	*11*.*5*
D1	57	50	1.1	21	36	0.4	0.7	66	33	29	381	109	280	222	610	0.6	0.4
D2	56	50	1.1	17	39	0.3	0.8	61	32	29	206	130	215	200	545	0.4	0.3
*Average*	*57*	*50*	*1*.*1*	*18*.*8*	*37*.*5*	*0*.*4*	*0*.*8*	*63*.*3*	*32*.*5*	*28*.*8*	*293*	*120*	*247*	*211*	*578*	*0*.*5*	*50*.*1*
*S*.*D*.	*0*.*7*	*0*.*0*	*0*.*0*	*3*.*2*	*2*.*1*	*0*.*1*	*0*.*0*	*3*.*9*	*0*.*8*	*0*.*0*	*124*	*15*	*45*	*15*	*46*	*0*.*2*	*17*.*4*

All distance measurements are in centimetres; angles are in degrees. N/A: Measurements could not be determined due to e.g., absence of digit impressions. Abbreviations: TL—track length; TW—track width; II^III, III^IV, II^IV—interdigital divarication angles of respective digits; te—toe extension whereby digit III projection length is past digit II and IV; (TL-te)—metatarsophalangeal length; MPL—metatarsophalangeal pad length; MSA- metatarsophalangeal surface area; SA—surface area. TL is the distance from the posterior margin of the track to the tip of digit III.

The Surface areas of all ichnites have been measured using ImageJ software on scale-calibrated photographs and figures. Using the freehand drawing tool, the perimeter of the track was outlined as accurately as possible. ImageJ then generated an area measurement for the outlined, irregular surface. This was done for the metatarsophalangeal area as well as for each individual digit (exclusive of claw mark impressions, which are differentially preserved). Individual digits’ surface areas were measured by using a straight line linking the hypex between digits II^III and III^IV as a base line. If the medial and lateral hypices were not on the same level, a straight line was drawn across from the higher hypex. The lateral hypex shows the most variability in theropod tracks [41] and therefore this method aims to avoid any pitfalls associated with this variability. The area below this line was treated as the metatarsophalangeal ‘heel’ pad region. This method, while not taking into account many morphological details, is a rough guide to estimate the proportions of ‘heel’ to digit area.

A digital model, using 2-D cartography and photogrammetry, was made for the large trackway surface from the site. Photogrammetric models were undertaken using a Canon PowerShot EOS D1200 (Focal length 28 mm, 5184 x 3456 resolution) following the methods provided in Mallison and Wings [42]. AgisoftPhotoscan (standard version 1.1.4) software was used to process point clouds. Three-dimensional models were converted to colour maps in the open source CloudCompare software (v.2.6.1, http://www.danielgm.net/cc/). Orthophotos were used for individual track D footprint images. Rubber silicon replicas were made of trackway D and are housed in the Ichnology Collection of the Evolutionary Studies Institute (ESI) at the University of Witwatersrand, South Africa (accession number: BP/6/735). All 3D surface models and their raw data are deposited at Figshare.

Calculations of hip height (*h*) and body length (*L*) from the tracks were made using Thulborn’s [43, 44] methodology, as adapted in Weems [45], for track lengths greater than 35 cm. Thulborn’s [44] morphometric and allometric ratios are as follows:

1. Hip heights (*h*) for theropods:
  $$h=3.06\times {\text{TL}}^{1.14}\left(\text{TL}<25-35\;\text{cm;}\;\text{allometric}\right)$$
  $$h=8.6\times {\text{TL}}^{0.85}\left(\text{TL}\geq \text{35}\;\text{cm;}\;\text{allometric}\right)$$
  h=4.5×TL(TL<25−35cm;morphometric)
  h=4.9×TL(TL≥35cm;morphometric)
2. Body lengths (*L*) for the theropod dinosaurs:
  L=4×h(TL<25−35cm)
  L=2×h+3.5(TL≥35cm)

Gait of the trackmaker for trackway A was measured and estimated by the ratio of stride length (*λ*) to hip height (*λ/h*). Dinosaurian gaits are classified as a “walk” (*λ/h*≤ 2.0), “trot” (2.0 < *λ/h* < 2.9) or “run” (*λ/h*≥ 2.9), using the approximation of Thulborn and Wade [46]. In calculating the gait the appropriate speed calculation could then be determined. Here, speed was calculated using Alexander’s [47] equation for walking gaits:

$$u=0,{25g}^{0,5}\times \lambda^{1.67}\times h^{-1.17}$$

Where, g = gravitational acceleration in m/sec, *λ* = stride length, and *h* = hip height (*h* = 8.6 × TL^0.85^).

## Results

### Sedimentology of the megatheropod trackway site

**Fig 2:**
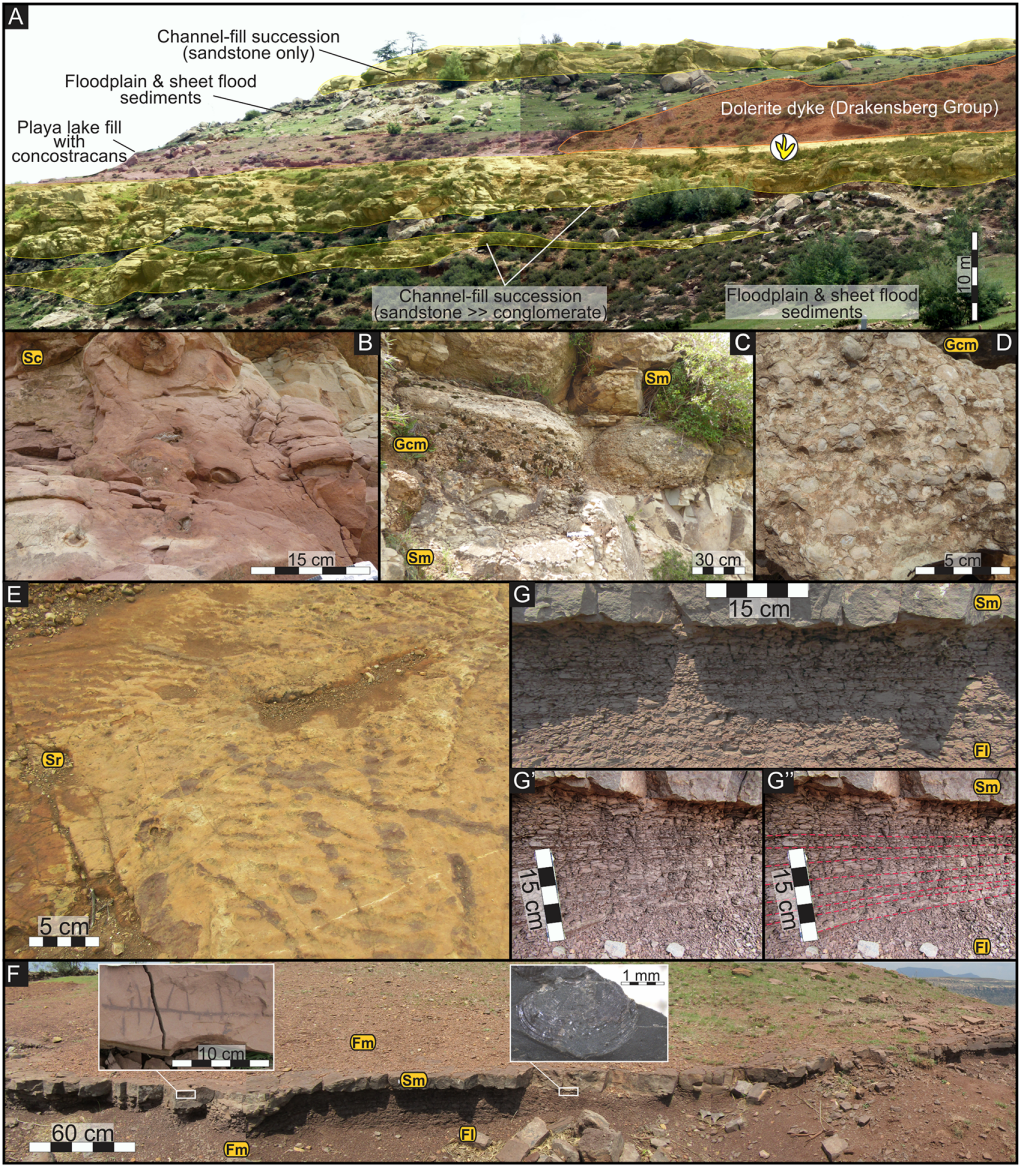
Sedimentological aspects of the Lower Jurassic semi-arid, fluvio-lacustrine upper Elliot Formation at Matobo in the Roma Valley. (A) The channel-like coarse-grained facies association forms multi-storey, upward-fining successions and is interbedded with the fine-grained facies association comprising mudstones and single-storey sandstones. See text for details. (B) Clast-rich sandstones (facies Sc) are regionally recurring and unique facies in the uEF. Note the clusters of in situ pedogenic carbonate nodules in facies Sc indicative of palaeo-pedogenic overprinting. (C and D) Close-up photographs of the channel-like, coarse-grained facies association, which here comprises massive sandstones and diagnostic, massive, clast-supported carbonate nodule conglomerate (facies Gcm). (E) Ripple cross-laminated sandstone bed at the top of an upward-fining succession terminates in asymmetrical ripple marks and bears the vertebrate tracks. (F and G) The fine-grained facies association contains, in addition to the dominant mudstones, single-storey, sheet-like, massive sandstones (facies Sm) with root traces (left inset). Here the sandstone overlies conchostracan-bearing (right inset), finely laminated mudstones (facies Fl) that display rhythmical bedding (see G, G’, G”). The former is indicative of sheet-floods, the latter is evidence for playa lake conditions in a seasonally wet, semi-arid floodplain setting.

The Elliot Formation in the vicinity of the megatheropod trackway site within the Roma Valley is a ~185 m thick succession of clastic sedimentary rocks, of which ~25 m belongs to the lower Elliot and ~160 m to the upper Elliot formations (Fig 1C). The former is exclusively exposed in patchy outcrops that are to the west of the trackway site and below the level of diagnostic carbonate nodule conglomerates (facies Gcm) of the uEF (Figs 1B and 2).

The uEF at Matobo can be subdivided into two major facies associations based on their shared characteristics of sedimentary features, geometries, lithology and grain size. The fine-grained facies association (Figs 1C and 2) is dominated by deep red, maroon to deep pink laminated and massive mudstones (facies Fl, Fm) that contain desiccation cracks, rootlets, and large, *in situ* carbonate nodules (Fig 2A, 2G and 2F). The laminated mudstones (facies Fl; Fig 2G) show rhythmical bedding and comprise silty mudstones and clay drapes with rare conchostracans (Fig 2F).

The mudstones are interbedded with fine-grained sandstone beds, which are either single storey (< 50 cm thick; Fig 2A, 2G and 2F) or multi-storey and form upward-fining successions that are up to 10 m thick (Figs 1C and 2A). The latter, forming the coarse-grained facies association at Matobo, is typically based by a ~25 cm thick, massive, poorly sorted, bone-bearing, reworked carbonate nodule conglomerate (facies Gcm–Fig 2C and 2D) that is laterally traceable in excess of 100 m. In this regionally recurring and unique conglomerate in the uEF [36, 37, 48], the nodules range from rounded to sub-angular, are poorly to moderately sorted, and are grey, white-to-red in colour. The clasts commonly form a clast-supported fabric (Fig 2D). The rest of the multi-storey sandstone package is dominated by very fine- and fine-grained sandstones (with subordinate medium-grained sandstones) that are either massive, with or without clasts (facies Sc, Sm; Fig 2B and 2C), or ripple cross-laminated (facies Sr) towards the top of the succession, where ripple marked surfaces are vertebrate track bearing (Fig 2E). The clast-rich sandstones (facies Sc; Fig 2B), another regionally recurring and unique rock type in the uEF [36, 37], is light pink or deep red, maroon and contains poorly sorted, 1–4 cm angular, rip-up mudstone clasts and localized, faint laminations. Locally, it may contain pedogenic carbonate nodules.

The overall geometry of the coarse-grained facies association is channel-like, whereas the interbedded mudstones and sheet-like, fine-grained sandstones are tabular and laterally persistent (Fig 2A). Based on local and regional sedimentological and palaeontological evidence (also see [36, 48]), the former is interpreted as ephemeral fluvial channel fills, whereas the latter as palaeo-pedogenically altered sediments of floodplains with sheet-flood deposits and shallow playa lakes. The former were pedogenically altered (rootlets), whereas the latter were temporarily inhabited by conchostracans and received seasonal sediment supply (rhythmical bedding) characteristic of fluvio-lacustrine settings under seasonally wet, semi-arid climatic conditions.

### Description of tracks and trackways

**Fig 3:**
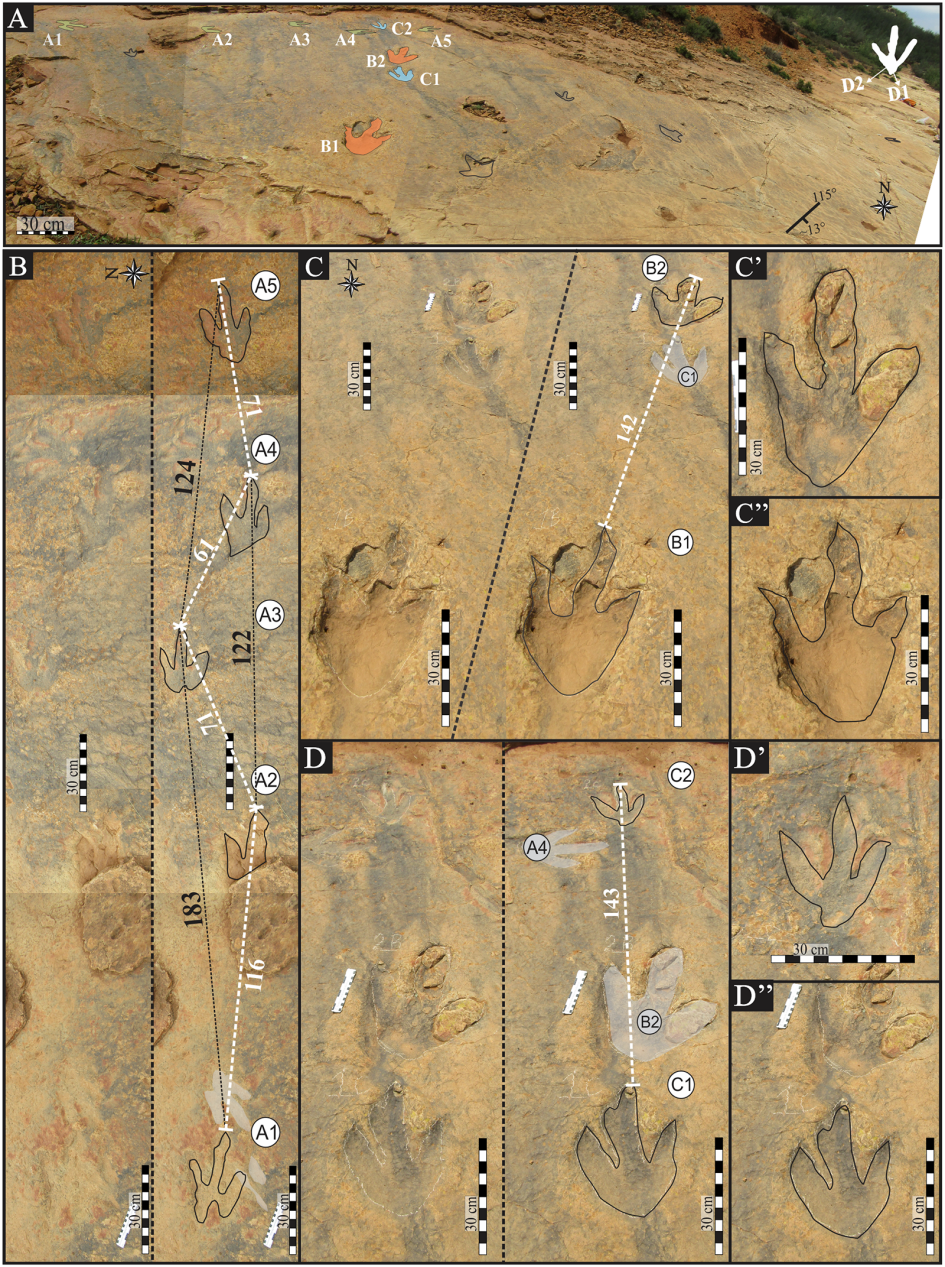
Photograph-based interpretive outline drawings of the tridactyl bipedal ichnites (trackways A, B and C) at the Matobo. (A) Overview of the individual tracks (not numbered) and four trackways (trackways A-D). (B) Matobo trackway A is 4 m long, runs in an east-west direction and consists of 5 consecutive ~32 cm long tracks. (C) Trackway B is perpendicular to Matobo A and is made up of two consecutive 40 cm long tracks, with stride length of ~1.4 m. Insets show (C’) right pes impression on more competent substrate and (C”) left pes impression on less competent surface (i.e. as determined from irregular outline of digits). (D) Trackway C is situated at the intersection of Matobo A and B and has a stride of 1.4 m. Insets show right (D’) and left (D”) pes impressions, and again with the right pes impression being made on an apparently more firm (competent) substrate. See Table 1 for more details.

**Fig 4:**
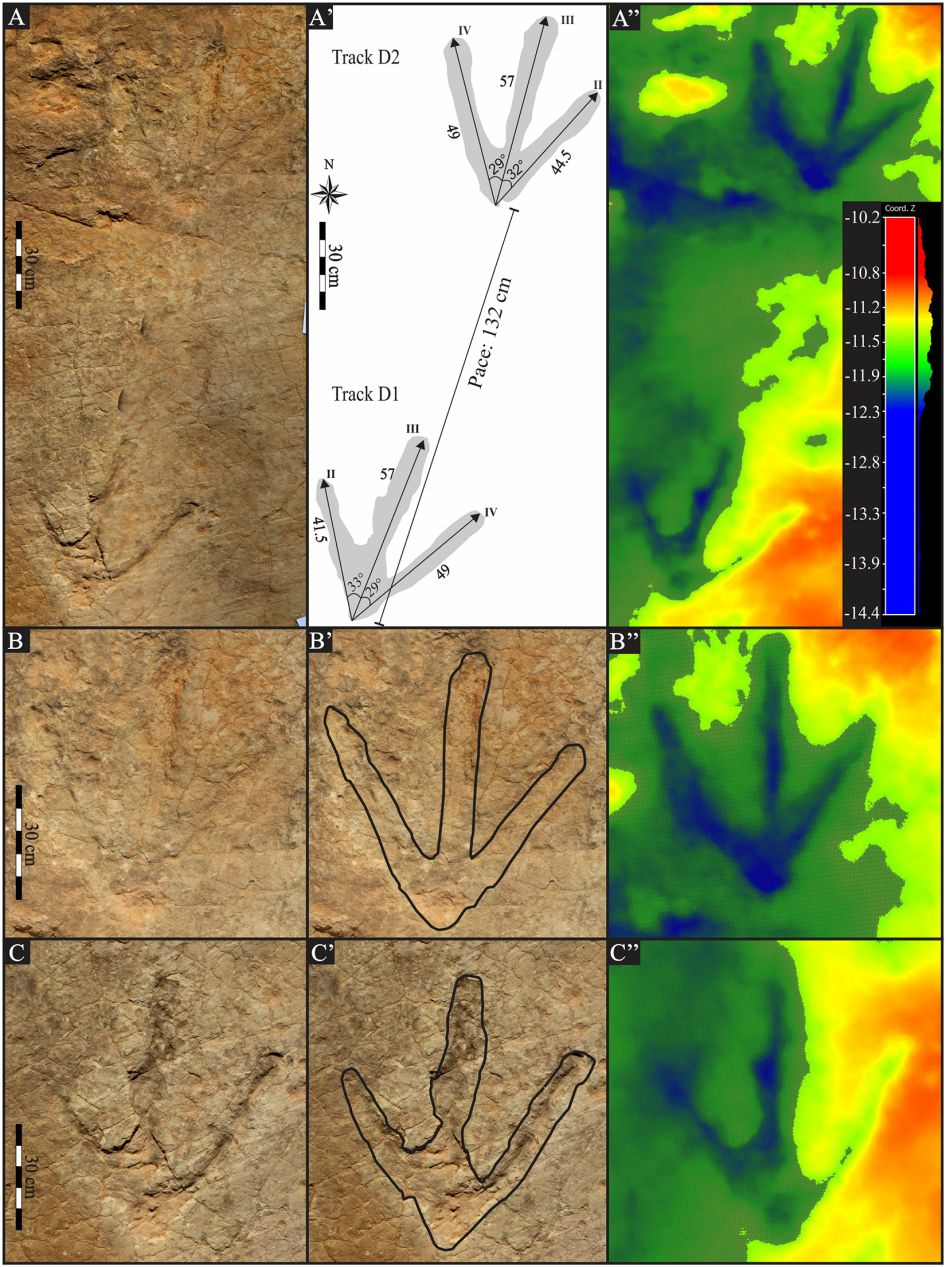
Photograph and interpretive outline drawings (A’—C’) with relevant measurements of Matobo trackway D left and right pes. (A”) False-colour depth analysis of trackway and (B”—C”) individual pes (highest topography is marked in red and lowest points in dark blue). The pes impressions are of three slender digits without digital pads impressions or claw marks. The rounded digits and the strongly V-shaped metatarsophalangeal margin had been lightly impressed on the palaeosurface. Distance measurements in cm; angles in degrees.

Approximately twenty tridactyl tracks, in varying degrees of preservation (preservation grade between 1 and 2; [49]), are present on the Matobo palaeosurface. The tracks appear to be both true tracks (with natural casts) and undertracks at the top of a sandstone bed. Of these tracks, eleven form four trackways (hereafter referred to as Matobo A, B, C, and D) and the remaining are discrete, scattered footprint impressions. These remaining nine tridactyl tracks are discussed in Ambrose (2003) and range in size between 30–35 cm. Matobo A consists of 5 tracks (Fig 3A and 3B) that are directed to the east, where as Matobo B, C (Fig 3C and 3D) and D (Figs 3 and 4) comprise of two consecutive tracks trending in a general north-south direction. All measurements are recorded in Tables 1 and 2. The left pes component of Matobo B and C (Fig 3C” and 3D”) is deeply impressed and potentially indicate a wet, less competent substrate in the southern portion of the palaeosurface (Fig 3). In contrast, at the northern extremity of the palaeosurface, Matobo A and, ~ 4 m to the west, Matobo D are shallowly impressed. The palaeosurface substrate consistency was highly variable over short distances and the size of the track and, by extension the animal, did not have bearing on the competency of the substrate. Unfortunately, the integrity of the Matobo palaeosurface is affected by traffic in the form of carts, livestock, and occasional minibuses, which use most parts of the surface as an informal road. This damage is manifested in the form of NE-SW running grooves that can be observed in photogrammetry models (see supplementary material and Fig 4).

**Table 2:** Measured trackway parameters (pace length, angulation, track length, stride) and estimated hip height, gait and speed of Matobo trackmaker of trackway A.

Matobo track #	Pace length (m)	PANG (°)	Matobo track #	TL (m)	Stride (m) λ	Morphometric hip height (m)	Gait	Speed (m.s-1)	Allomertric hip height (m)	Speed (m.s-1)
A1-A2	1.0		A1-A3	0.33	1.8	1.5	1.2	1.3	1.6	1.2
A2-A3	0.6	155	A2-A4	0.31	1.2	1.4	0.9	0.7	1.5	0.7
A3-A4	0.4	139	A3-A5	0.31	1.2	1.4	0.9	0.8	1.5	0.7
A4-A5	0.5	159								
**Average**	0.6	151.0		0.3	1.4	1.4	1.0	1.0	1.6	0.8
**S.D.**	0.3	10.6		0.0	0.3	0.1	0.2	0.3	0.1	0.3

Measurements are in metres and degrees. Abbreviations: PANG—pace angulation; TL—track length; S.D.—standard deviation.

Tracks of Matobo A, B and C are moderately large in size (TL: 30–40 cm), tridactyl, digitigrade and elongate (TL/TW = 1.2–1.5) with weak to moderate mesaxony (av. 0.5; based on the anterior triangle ratio l/w of Lockley [49]; Table 1; Fig 3). The digits are transversely thick relative to their anteroposterior length, tapering to V-shaped tips, and are fairly straight but show slight divergent curvature along the tips of digits II and IV. They lack digital pad impressions and rarely preserve pointed claw marks (e.g. tracks A2, B1; Fig 3). High divarication of digits II^IV (56°–58°) is notable and the interdigital divarication angle II^III (27°–30°) is marginally larger than III^IV (28°–29°; Table 1). V-shaped hypices between digits are noted (Fig 3B, 3C and 3D). The heel margin, which is the metatarsophalangeal pad impression of the foot, is rounded and U-shaped in Matobo B and to a lesser extent in Matobo A (more V-shaped). Matobo C’s posterior margin is more V-shaped in appearance.

Matobo A is a 4 m long trackway comprised of 5 consecutive tracks (Fig 3B). The preservation of the tracks along the extent of the trackway is relatively consistent. The tracks have an average length (TL) and width (TW) of 32 and 23 cm, respectively, giving an average TL/TW ratio of 1.4. Tracks appear more gracile and digitigrade than Matobo B and C on the same surface, but this appears to be largely a function of substrate firmness. The trackway is narrow with the pace angulation (PANG) ranging between 139° and 155°. The pace length varies between 0.4 m and 1.0 m with a general decrease in pace length from track A1 to A5 (west to east; Fig 3B). Accordingly, the stride length decreases between track A1 and track A5 from 1.8 m to 1.2 m (Table 2). The speed of the animal, based on the calculated morphometric hip height, shows a corresponding deceleration from 1.2 ms^-1^ at the start of the trackway to 0.7 ms^-1^ at the end of the trackway (Table 2).

Matobo B consists of two tracks still containing some of their natural sandstone casts (Fig 3; Table 1). Tracks B1 and B2 (Fig 3C) have an average length and width of 40 cm and 32 cm, respectively, and a TL/TW ratio of 1.2 (Table 1). These represent the second largest tridactyl theropod tracks, to date, in the Elliot Formation at ~40 cm long (Fig 3C; Table 1). The digits appear relatively straight with pointed tips; however, the natural casts obscure the latter and prevent more accurate morphological observations. This may have also contributed to the exaggerated length and width of these tracks relative to Matobo A and C. Morphological detail (and hence preservation) of the right pes impression B2 (Fig 3C’) is better, as noted by the defined and relatively undistorted nature of the digits, than that of the right pes (B1; Fig 3C”). This appears as a function of the competency of the substrate.

The two tracks of Matobo C have an average TL and TW of 31 and 21 cm, respectively, giving a TL/TW ratio of 1.5. Morphologically, the tracks fit into the abovementioned general description of the Matobo site tracks and have no additional/unique features besides the more V-shaped appearance of the posterior margin. As previously noted, the right pes impression (Fig 3D’) shows is better preserved with no distortion of the digits.

Very large, tridactyl, digitigrade, right (D1) and left (D2) pes tracks are situated in the easternmost sector of the Matobo palaeosurface (Matobo D; Figs 3 and 4). The average track length and width is 57 cm and 50 cm, respectively, and the TL/TW ratio is 1.1 (Table 1). The tracks show weak to moderate mesaxony with a calculated mesaxonic index of 0.5. Digit impressions are shallowly depressed (Fig 4B and 4C). Digits are straight with rounded ends and lacking claw marks, although the photogrammetry images (Fig 4A”, 4B” and 4C”) show more pointed digits tips than can be observed in the field (or from the line drawing). The digits do not taper but maintain a fairly uniform width along their length. There is no hallux impression. The length of digit II is 41.5 cm in D1 and 44.5 cm in D2, thus digit II is shorter than digits IV (49 cm long), and both are shorter than digit III (57 cm long). The free length of digit IV is greater than digit II because the hypex of digit IV is lower than the medial hypex. The ratio of the posterior margin of the heel to the base of the hypex of digit II and digit IV averages 2.3 and 3.2 for D1 and D2, respectively. Interdigit angles have low variability between tracks with an average of 33° and 29° for II^III and III^IV, respectively (Table 1). Total divarication (II^IV) is 66° in D1 and 60.5° in D2.

There are no digital (phalangeal) pads impressions preserved and the metatarsophalangeal ‘heel’ pad forms a distinct, small, semi-circular impression (Fig 4B and 4C). The ‘heel’ margin is V-shaped. In both tracks, this depression is 17 mm deep proximally and shallows to 14 mm towards the distal digit margin and tip of digit III (Fig 4B” and 4C”). The depth of the ‘heel’ and digits is slightly irregular (Fig 4B” and 4C”), and the depth of digits III and IV appear deeper than digit II.

#### Intra-track variation at Matobo

**Fig 5:**
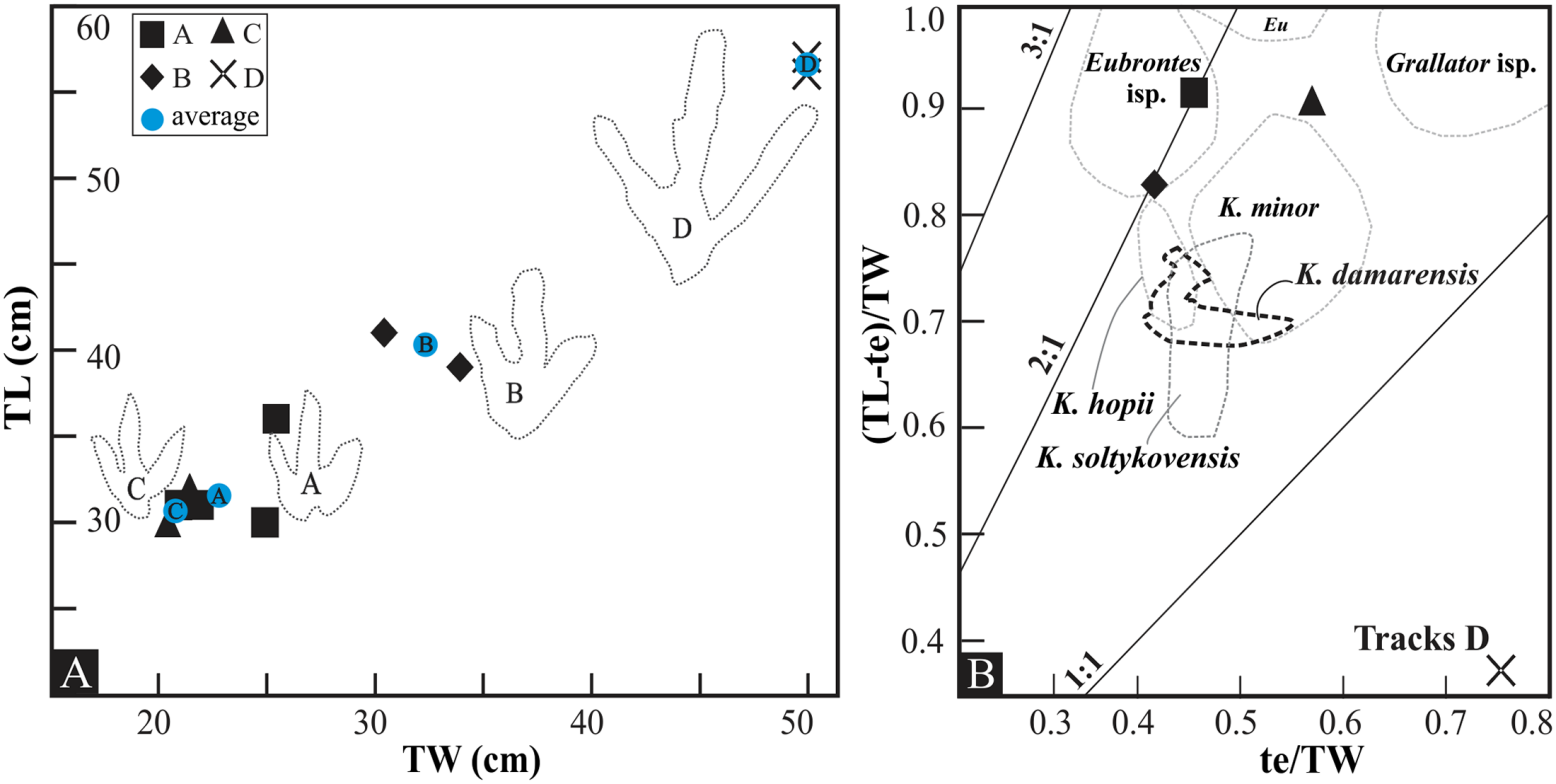
Bivariate plots illustrating morphological variability. (A) Intra-track morphological variability in track length (TL) and width (TW) of the four tridactyl Matobo trackways (labelled A–D). Outlines of the tracks are shown for visual contrast. (B) Averaged proportions of the Matobo tracks and Lower Jurassic tracks mentioned in Weems [50] using the (TL-te)/TW versus te/TW ratios. Demarcated fields are for *Eubrontes* isp., *Kayentapus minor*, *Kayentapus hopii* and the Southern hemisphere *Kayentapus damarensis* [51]. The lines labelled ‘3:1’, ‘2:1’ and ‘1:1’ denote the ratio of digit III extension of either one-third, one half or equal to metatarsophalangeal length [50]. Abbreviations: TL—foot length, TW—foot width, te—toe extension: digit III projection length past digit II and IV.

Bivariate morphological analyses, as presented in Fig 5A, show moderate morphological intra-track variability in Matobo A, B and C (Table 1; Figs 3 and 5). This is a phenomenon commonly related to the rheology of the substrate, which is controlled by moisture content and sediment properties (e.g., grain size, sorting). Intra-track variability is low for pes pairs from Matobo B, C, and D despite the perceived changes in the substrate consistency (Figs 3C, 3D and 4A). Conversely, the variability observed in Matobo A (Fig 5A) is more likely related to the slowing gait of the animal (from 1.2 to 0.7 m s^-1^ over a distance less than 4 m; Fig 3; Table 2) than to the substrate, which shows no marked changes in consistency along the length of the trackway.

Fig 5B illustrates the morphological ratios of the Matobo tracks and the bounding ranges of well-known Lower Jurassic tracks [50]. The imprecise placement of Matobo A-D within the comparable North American ichnotaxa fields (Fig 5B) is likely related to differences in the Gondwana forms versus these Laurasian ichnotaxa.

The averaged values for Matobo C plot outside of the ranges of *Eubrontes*, *Kayentapus hopii*, and *Kayentapus damarensis* [51], and closer to the field of *Kayentapus minor* (Fig 5B). Conversely, averaged values for the Matobo A and B plot within the range given for *Eubrontes* isp. but as outliers on the ratio line ‘2:1’ (digit III extends half the metatarsophalangeal length) (Fig 5B). The U-shaped posterior metatarsophalangeal margin (hereafter referred to as the ‘heel’) typical of *Eubrontes* is seen in Matobo B and this lends support for their placement within the *Eubrontes*-range, but less so with Matobo A. Additional shared features of Matobo A and B with this ichnotaxon are their moderately large size (av. 34 cm), average TL/TW of 1.2–1.4, weak to moderate mesaxony (av. 0.5; Table 1), broad digits (digit II and IV being approximately subequal in length), and claw marks preferentially preserved. Conversely, Matobo C, while sharing several similar gross morphological features (e.g., broad, thick digits,) with Matobo A and B (Fig 5A), is morphologically closer to *Kayentapus*-like tracks (Fig 5B). It displays a wide divarication and a more V-shaped heel typical of *Kayentapus*-like tracks [52, 53]. Matobo A also displays some *Kayentapus*-like traits with respect to its narrow pace angulation (139°–155°) and large stride length (1.8 m–1.2 m; Table 2), which are similar to the type material of *K*. *hopii* (pace angulation: 174°, stride: 1.8–1.9 m; [53]). In general, the wider II^IV divarication angles (av. 57°) of Matobo A, B, and C are greater than the range and uppermost limit of *Eubrontes* (40°; [54]). Wider divarication is common to *Kayentapus*-like tracks [53] and a wide II^IV divarication angle (62°) has been reported for other Lesotho tridactyl tracks, for instance *Neotrisauropus deambulator* Ellenberger [54] (UEF, Moyeni, Quthing District), which is an ichnite that has been designated to *Kayentapus* by Piubelli et al. [52].

In contrast to Matobo A–C, Matobo D is significantly longer (by 17–25 cm), with narrower digits, a slightly wider total (II^IV) divarication angle (63°), lower TL/TW (1.1 versus 1.4–1.5 range for *Eubrontes*; Table 2) and a V-shaped posterior margin. Despite the low TL/TW ratio (1.1), which falls below the reported threshold values of >1.25 for theropods [55], this trackway is considered of theropod origin due to a suite of other morphological characteristics. Fig 5B demonstrates that Matobo D plots away from all parameter fields of *Kayentapus* and *Eubrontes* and below the ratio line of 1:1, with a (TL-te)/TW of 0.4 and te/TW of 0.8. Matobo D and other large, globally occurring taxa (ratios presented in Tables 2 and 3) do not conform to Weems’ [50] foot measurement ratios specifically because their (TL-te)/TW are very low. The pronounced lily-shaped/V-shaped posterior margin of tracks D is particularly notable and similar to *Kayentapus* isp.

**Table 3:** Measurements and ratios of various Lower Jurassic, Middle Jurassic and Upper Cretaceous theropod tracks and those of the current study at Matobo (tracks A–D).

Track	N	Reference	Age	(TL-te)	te	TL	TW	Interdigit angle			Ratios				Surface area (SA) cm^2^					
								II^III (°)	III^IV (°)	II^IV (°)	TL/TW	(TL-te)/TW	te/TW	MPL/TL	MSA	Digit II	Digit III	Digit IV	Total digit SA	MSA/total digit SA
Matobo tracks A	5	this study	Lower Jurassic	11.5	20.5	31.8	23.4	28.2	28.1	56.2	1.4	0.5	0.9	0.4	130.3	47.9	83.5	52.9	184.4	0.7
Matobo tracks B	2	this study	Lower Jurassic	17.5	22.5	40.0	32.3	26.5	29.3	56.9	1.2	0.6	0.7	0.4	357.2	78.7	158.7	59.1	296.5	1.5
Matobo tracks C	2	this study	Lower Jurassic	12.0	19.0	31.0	21.0	29.7	28.7	58.4	1.5	0.6	0.9	0.4	133.9	57.1	101.6	69.1	227.7	0.6
Matobo tracks D	2	this study	Lower Jurassic	19.0	38.0	57.0	50.0	32.5	28.8	63.3	1.1	0.4	0.8	0.3	358.6	130.2	285.0	226.0	641.2	0.6
*Irenesauripus mclearni*	1	72	Upper Cret.	17.0	31.0	48.0	37.0	39.0	33.0	72.0	1.3	0.5	0.8	0.4	233.0	118.4	169.8	99.6	387.8	0.6
*Irenesauripus acutus*	1	76	Lower Cret.	21.0	32.5	53.5	40.0	18.0	40.0	58.0	1.3	0.5	0.8	0.4	263.0	107.0	153.9	130.9	391.8	0.7
*Irenesauripus*	1	71	Upper Cret.	23.0	30.0	53.0	43.1	35.0	29.0	64.0	1.2	0.5	0.7	0.4	400.0	216.0	278.0	240.0	734.0	0.5
*Eubrontes* (?) *glenrosensis*	1	67	Cretaceous	45.0	19.0	64.0	48.0	27.0	29.0	57.0	1.3	0.9	0.4	0.7	818.0	86.0	278.0	141.0	505.0	1.6
*Eubrontes* cf. (right)	1	66	Middle Jurassic	20.0	26.0	46.0	31.9	30.0	28.0	58.0	1.4	0.6	0.8	0.4	272.4	75.0	142.2	116.4	333.6	0.8
*Eubrontes* cf. (left)	1	66	Middle Jurassic	25.0	24.0	49.0	35.0	23.0	27.0	50.0	1.4	0.7	0.7	0.5	433.7	91.4	166.4	71.2	329.0	1.3
*Eubrontes giganteus*	1	55	Triassic-Jurassic	13.0	18.0	31.0	21.0	21.0	25.0	46.0	1.5	0.6	0.9	0.4	171.6	47.8	114.1	58.6	220.5	0.8
*Gigandipus*	1	69	Lower Jurassic	16.0	19.0	33.0	22.0	24.0	21.0	45.0	1.5	0.7	0.9	0.5	214.5	66.1	93.0	82.7	241.8	0.9
*Kayentapus hopii*	1	68	Triassic-Jurassic	6.0	19.0	29.7	26.0	35.0	29.0	64.0	1.1	0.2	0.7	0.2	22.9	48.8	103.4	58.7	210.9	0.1
cf. *Megalosauripus* isp.	1	61, 63	Lower Jurassic	17.0	29.0	49.0	35.0	22.0	34.0	56.0	1.4	0.5	0.8	0.3	356.8	143.3	305.0	170.9	619.2	0.6
*Kayentapus minor*	1	62	Triassic-Jurassic	15.0	24.0	39.0	28.1	30.0	30.0	60.0	1.4	0.5	0.9	0.4	220.2	59.6	121.5	54.1	235.2	0.9
*Eubrontes* isp.	1	62	Triassic-Jurassic	16.3	19.0	35.3	22.4	26.0	22.0	48.0	1.6	0.7	0.8	0.5	252.3	48.6	112.4	62.8	223.8	1.1
Large theropod Poland	1	62, 74	Triassic-Jurassic	35.9	18.4	54.3	37.7	22.0	33.0	55.0	1.4	1.0	0.5	0.7	337.7	184.5	227.7	190.0	602.2	1.8

Abbreviations: as per Table 1; N refers to the number of tracks measured. Distance measurements in cm; angles in degrees. This data was obtained from measurements of scaled photos taken from publications cited.

### Comparative ichnology

The V-shaped posterior margin of *Kayentapus* is considered to be one of its more distinguishing attributes [53], and is noted to occur in the Early Jurassic and then again in the Early Cretaceous as represented by the theropod ichnite *Irenesauripus* Sternberg [56]. In addition to the Northern Hemisphere occurrences, *Kayentapus* has been described from Madagascar [57] and Namibia [51, 58], but has not been formally recognised in Lesotho. Piubelli et al. [52] have considered that several ichnotaxa from Lesotho, namely *Deuterotrisauropus socialis*, *Kleitotrisauropus moshoshoei* (originally *Kainotrisauropus moshoshoei* in Ellenberger, [20]), *Neotrisauropus deambulator*, and *Neotrisauropus leribeensis* may be synonymous with *Kayentapus*. This was reaffirmed by Lockley et al. [53], whereas originally Olsen and Galton [30] had synonymised *Kleitotrisauropus*, *D*. *socialis*, and *N*. *deambulator* with *Grallator*.

*N*. *deambulator* and *N*. *leribeensis* display the high total (II^IV) divarication angles (62° and 48°, respectively) and greater III^IV than II^III divarication angles which are attributes of *Kayentapus*. *D*. *socialis* and *Kleitotrisauropus* have TL, TW, and interdigital divarication that are within the range of, and therefore comparable to, both *Kayentapus* and *Eubrontes*. *D*. *socialis*, however, is Late Triassic in age as it occurs at Maphutseng and Subeng [20], both of which are lower Elliot Formation sites. *Kleitotrisauropus* (*Kainotrisauropus*) *moshoshoei* occur at the Matsieng, Qalo, and Matelile localities [20] and have not been viewed by the current authors (due to inaccurate locality information), but are considered Early Jurassic in age (zone B/5 in Ellenberger, [24]). In fact, Ellenberger [24] suggested that *Kleitotrisauropus* (*Kainotrisauropus*) is comparable with *Eubrontes* of Lodève in France. In contrast, the ichnospecies *Kainotrisauropus morijiensis* was noted by Ellenberger [20] as reminiscent of *K*. *minor* of the Hettangian in France. A formal revision of these ichnotaxa is needed to formalise their potential assignment to *Eubrontes*, *Grallator*, or *Kayentapus*

Similarities between *Eubrontes* and *Kayentapus* isp. have been noted in the southern African/ Gondwana tracks described by Wagensommer et al. [51]. The Jurassic Namibian tracks reported in their study are medium to large (range of FL = 25–35 cm, FW = ~13–25 cm), have robust digits, moderate total divarication (~40°; in line with expected range of *Eubrontes*), and interdigital angles between II^III = 10–15° and III^IV = 25–30°. Wagensommer et al. [51] designated tracks ONP VII_1 to *Eubrontes giganteus* and ONP I_1 to *Kayentapus damarensis*. The latter tracks (ONP I_1) were assigned to *Kayentapus* because, in comparison to *Eubrontes*, the greater toe extension and shorter TL/TW ratio were considered diagnostic of that ichnogenus with respect to Weems’s [50] foot measurement ratios. However, as noted by Wagensommer et al. [51], this does not take into account other features such as the divarication angle, robustness of the digits, or stride. Weems [50] argued that these characteristics are difficult to consistently measure because of variation relating to either pace or substrate. The underlying issue, however, relates to the homogeneity of theropod dinosaur foot morphology globally during this time. Thus, the lack of marked differentiation between ichnotaxa is potentially a reflection of gross similarities in foot morphology and the generalised manner of bipedal locomotion typical of Early Jurassic theropods [59].

In the current study, the Matobo tracks also present evidence for and against their assignment within both *Eubrontes* and *Kayentapus*. Again, this seems to trend with other southern African tridactyl tracks which do not completely conform to Northern Hemisphere standards. Despite the suggested affinities to *Eubrontes* for Matobo A and B within Weems’ [50] scheme, their higher II^IV divarication angle, moderate mesaxony, and V-shaped posterior margin also suggests a possible referral to *Kayentapus*. The latter possibility is further supported by comparable morphological rations (te/TW and {TL-te)/TW}). However, the evidence against placement within *Kayentapus* rests on the divarication between digits III and IV equal to or less than between digits II and III, and the significantly larger FL and FW.

Track B is considered as being more *Eubrontes*-like due to its significantly U-shaped heel margin in combination with its large track size and robust digits (Figs 3C and 5). In contrast, the dimensions of Matobo trackway A are broadly consistent with those of *K*. *minor*, while the V-shaped heel and wide divarication of both track A and C (features not taken into account in the plot of Fig 5B) also suggest a possible referral to *Kayentapus* (Fig 5A). As previously discussed, there are several globally recognised Late Triassic-Early Jurassic theropod ichnotaxa: *Eubrontes*, *Gigandipus*, *Anchisauripus* (synonymised by most with *Grallator*), *Grallator*, and *Kayentapus*. While the size of the track is by no means a distinguishing factor, none of these ichnotaxa are wholly comparable to Matobo D, although the closest morphological affinities are seen in the ichnogenus *Kayentapus*. Thus, for the ostensible purpose of classification, Matobo D is considered here as *Kayentapus*-like, although its relationships to other large, globally occurring ichnites is given further exploration in the comparative ichnology discussion below. In summation, despite the wide II^IV divarication angle for Matobo A–D and the relative symmetrical distribution of the interdigital angles II^III and III^IV, the morphological evidence presented above (digit width, TL, TW etc.; Table 1 and Fig 5) places at least Matobo A and B into the ichnogenus *Eubrontes* (Fig 5B) and Matobo C and D as belonging to a *Kayentapus*-like animal.

### Comparative ichnology with large tracks

**Fig 6:**
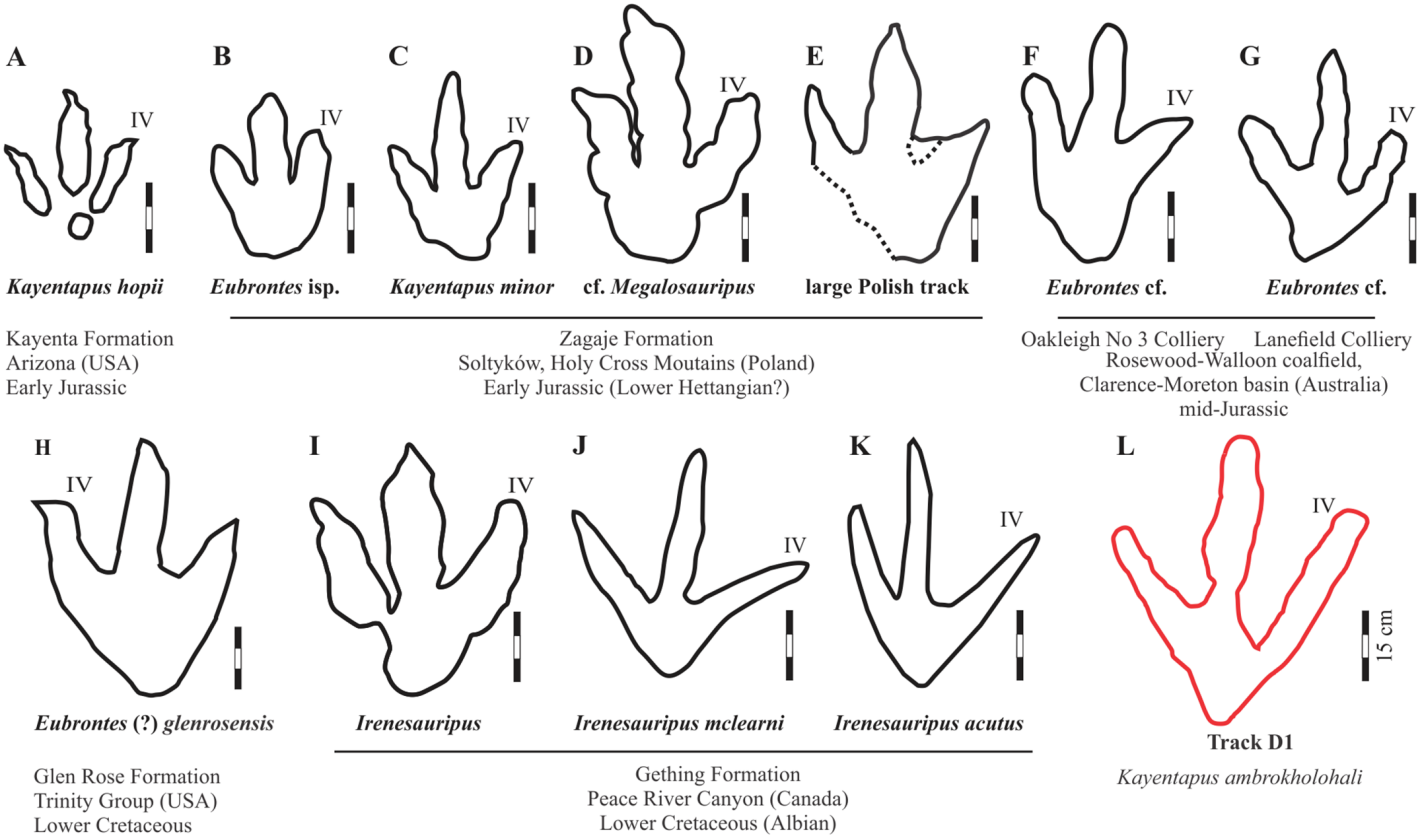
Comparative line drawings of Lower Jurassic track D1 at Matobo and other large theropod tracks from the Jurassic and Cretaceous. (A) *Kayentapus hopii*, Kayenta Formation (early Jurassic [67]). (B) 35 cm long *Eubrontes* isp. (C) 39 cm long *Kayentapus minor*. (D–E) cf. *Megalosauripus* (Muz. PIG 1661.II.1) and large Polish theropod track from the Sołtyków site, Poland [60, 61]. (F–G) *Eubrontes* cf., from the middle Jurassic of Australia [65]. (H) *Eubrontes* (?) *glenrosensis*, Lower Cretaceous Glen Rose Formation (USA) [66]. (I) *Irenesauripus* in Lockley et al [67]. (J) *Irenesauripus mclearni* McCrea et al. [71]. (K) *Irenesauripus acutus* in McCrea [75] all occuring within the Albian Gething Formation (Canada). (L) Track D at Matobo (*Kayentapus ambrokholohali*) of this study. All images were redrawn and scaled to 15 cm. See text and Table 3 for details.

Matobo A–D are broadly assessed against other large, valid ichnotaxa, irrespective of age differences of up to 100 million years or geographical occurrence (Tables 3 and 4). Matobo D represents a unique megatheropod trackway in the Early Jurassic. There are no Early Jurassic theropod tracksites of comparable size excepting several large footprints reported from the uppermost part of the Sołtyków outcrop of Poland (presumed Hettangian in age; Tables 3 and 4; Fig 6 [60, 61]). One of the first described gigantic Polish tracks (Muz. PIG 1661.II) was studied by Gierliński et al. [60], and is reported as 54 cm long, with robust digits, claw impressions and, uniquely, a large metatarsophalangeal area constituting 33% of foot length. Gierliński et al. [62] suggested this track was most similar to Upper Jurassic theropod footprints (i.e. *Megalosauripus*; [63, 64]) based on the large metatarsophalangeal area. Three other large tridactyl forms are reported from the Sołtyków site (39 cm TL *Kayentapus minor*; [61]; 35 cm TL *Eubrontes* isp; [61]; 50–65 cm TL large theropod footprints e.g. MPT.P/146 [61]) and are presented here (Table 3).

**Table 4:** Track makers body length (L) calculation from track length (TL) data.

Track	Ref	N	TL	Allometric hip height, *h*	Morphometric hip height, *h*	Body length (m)		
						Allometric body length	Morphometric body length	Averaged body length
Matobo A	this study	5	32	1.6	1.4	6.3	5.7	6.0
Matobo B	this study	2	40	2.0	2.0	7.5	7.4	7.4
Matobo C	this study	2	31	1.5	1.4	6.1	5.6	5.9
Matobo D	this study	2	57	2.7	2.8	8.8	9.1	9.0
*Irenesauripus mclearni*	71	1	48	2.3	2.4	8.1	8.2	8.2
*Irenesauripus acutus*	75	1	54	2.5	2.6	8.6	8.7	8.7
*Irenesauripus*	72	1	53	2.5	2.6	8.5	8.7	8.6
*Eubrontes* (?) *glenrosensis*	66	1	50	2.4	2.5	8.3	8.4	8.3
*Eubrontes* cf. (right)	65	1	46	2.2	2.3	8.0	8.0	8.0
*Eubrontes* cf. (left)	65	1	49	2.4	2.4	8.2	8.3	8.3
*Eubrontes*	54	1	31	1.5	1.4	6.1	5.6	5.9
*Gigandipus*	68	1	33	1.6	1.5	6.6	5.9	6.3
*Kayentapus hopii*	67	1	30	1.5	1.3	5.8	5.3	5.6
cf. *Megalosauripus* isp.	60, 62	1	49	2.4	2.4	8.2	8.3	8.3
*Kayentapus minor*	61	1	39	1.9	1.9	7.4	7.3	7.3
*Eubrontes* isp.	61	1	35	1.8	1.7	7.1	7.0	7.0
Large theropod Poland	61, 73	1	54	2.6	2.7	8.6	8.8	8.7

Hip height and body length measurements are in metres; track length (TL) is provided in cm; N refers to the number of tracks measured; if more than one track was measured from the same trackway, TL is an average value. Allometric body length is calculated from allometric hip height, and morphometric body length is calculated from morphometric hip height.

There are several commonly occurring tridactyl taxa in the Early Jurassic, with *Eubrontes giganteus* representing one of the larger forms (≥ 25cm; [54]). Other large (> 40 cm) *Eubrontes*-like tracks are reported from the Middle Jurassic (cf. *Eubrontes*, from the Rosewood-Wallon coalfield, Australia; Fig 6 [65]) and Lower Cretaceous (*Eubrontes* (?) *glenrosensis*, Glen Rose Formation, Trinity Group, USA; Fig 6 [66]). *Eubrontes* and large *Eubrontes*-like tracks (e.g. [54, 61]) typically have a TL/TW ratio of 1.4–1.5, digit impressions that are thick and sturdy, a short digit III extension and digits II and IV which project equally far along the axis of digit III [8, 54]. The total divarication angles between 25°– 40° [54]. When compared to very large *Eubrontes* tracks, Matobo D shares only a similar te/TW (0.8; specifically *Eubrontes*, *Eubrontes* isp. and *Eubrontes* cf.; [54, 61, 65]), with *Eubrontes*-like tracks bearing higher TL/TW ratios and a lower divarication angle (II-IV; 46° vs 63°). *Eubrontes* (?) *glenrosensis* ([66]), the Middle Jurassic Australian cf. *Eubrontes*, and Matobo D share comparable metatarsophalangeal length to total TL ratios.

*Kayentapus* (~35 cm TL; Table 3, Fig 6) is a gracile tridactyl track with a comparable age distribution to the Matobo tracks. This ichnogenus has been variously considered an independent ichnogenus (wider digit divarication and overall pace; [67]), ‘lumped’ within the *Grallator-Eubrontes* spectrum [54, 68], or considered synonymous within *Eubrontes* [8, 69]. Milner et al. [68] considered *Kayentapus* valid with respect to the divarication of digit IV and the length of projection of digit III. Interestingly, *Kayentapus* shows considerable variation both between tracks and within a single trackway, making an explicit morphological diagnosis for this ichnogenus difficult [64, 68]. Lockley et al. [70] have further recognised the ‘*Kayentapus*–*Magnoavipes’* morphotype, which identifies large theropod tracks (TL ≤ 35 cm) with very high divarication angles of 90° to 100°. Matobo D does not show this morphological characteristic, nor is there marked variability between tracks—although the latter may be accounted for by small sample size (N = 2). However, Matobo D, *Kayentapus hopii*, and *Kayentapus minor* (Table 2) share the V-shaped posterior margin, relatively slender/gracile digits, and comparable TL/TW ratio, although the latter (*K*. *minor*) is smaller in size with respect to both the TL and TW. The interdigital angles are very similar between these tracks (Table 3), with divarication angles of Matobo D (II^III = 33°, III^IV = 29°, II^IV = 63°) being similar to the Polish specimens of *Kayentapus* [61] and the North American *Kayentapus hopii* [67]. Dissimilarities are present with *K*. *hopii* when looking at the morphological (TL-te)/ TW and te/ TW ratios (Fig 5; Table 3). However, Matobo D’s (FL-te)/TW ratio of 0.4 is more comparable with *K*. *minor* (0.5) than *K*. *hopii* (0.2) [70, 71].

The ichnogenus *Megalosauripus* is a common Late Jurassic-Cretaceous form hypothesised to represent the megalosaurid (Tetanurae) dinosaurs dominant in this time period [63, 72]. This ichnogenus is not considered to occur prior to the Late Jurassic [60]. In comparing Matobo D to the Lower Jurassic (~Hettangian) Polish representative, tentatively cf. *Megalosauripus* isp. (and ‘giant polish theropod’; Tables 3 and 4; [60, 62, 73]), similarities are mainly noted in the TL (cf. *Megalosauripus* isp. of 49 cm and 54 cm with claw impression), track measurement ratio of (FL-te)/TW (metatarsophalangeal region/track width), phalangeal length/total TL ratios and total divarication angle (Table 3). However, despite some morphological parallels, the considerably less robust and distinct lack of a hallux impression set Matobo D apart from this Polish ichnotaxon. That said, a hallux impression is more likely a reflection of the rheology of the substrate than a function of the particular walking gait or style, especially given the generalised foot anatomy of basal theropods.

*Irenesauripus* (Gething Formation, Canada; Fig 6 [70, 74, 75]) is a Lower Cretaceous (Albian) ichnogenus that, despite its much younger age, shows strong similarities to Matobo D. There are two ichnospecies for this ichnogenera: *I*. *mclearni* and *I*. *acutus*, with a variable TL (28–53 cm) and divarication between II^III = 18°–39° and III^IV = 37°–40° (Table 2; [71, 75]). In particular, the following parameters are comparable between track D and *Irenesauripus* isp.: (a) TL/ TW ratio (1.2–1.3; Table 3); (b) II^IV divarication; (c) metatarsophalangeal length: total TL ratios; and (d) greater free length of digit IV than digit II. Furthermore, the track measurement ratio (FL-te)/TW (0.4) is comparable with both *I*. *mclearn* and *I*. *acutus*, while the te/TW (0.8) fits well with that of *Irenesauripus* [70, 71, 74, 75]. Digital pad impressions, absent in Matobo D, were not originally diagnosed for *Irenesauripus*, but this was later amended by Lockley et al. [70] on inspection of the type material. While Matobo D shares many morphological similarities with the ichnospecies *I*. *mclearni*, the total track surface area to TL ratio of the former is greater (1:18.5 cm^2^ vs 1:8.4 cm^2^ in *I*. *mclearni* calculated by McCrea et al. [71]), whereas its digit III^IV divarication angle is smaller (Table 3).

In summation, although Matobo D can be shown to be morphologically distinct from *Eubrontes* and *Eubrontes*-like tracks, distinguishing the former from either *Kayentapus* or *Irenesauripus* is much more problematic. However, despite the noted morphological similarities between Matobo D and the Cretaceous *Irenesauripus*, their great age disparity suggests that these similarities are primarily due to convergence. Matobo D is therefore interpreted as representing either an especially large variant within the *Kayentapus* morpho-spectrum, or a novel ichnotaxon. We consider it here, based on the morphological description provided, to be a much larger variant of the North American ichnogenus *Kayentapus* and as such we assign it to the ichnogenus *Kayentapus*. However, given it unique size and morphological traits (i.e. greater free length of digit IV vs. II) we hereby establish a new ichnospecies.

#### *Kayentapus ambrokholohali* ichnosp. nov., Fig 4

Diagnosis: two very large, 57 cm long and 50 cm wide, gracile tridactyl pes impressions with wide total divarication (II^IV) is 63°. The metatarsophalangeal pad of digit IV is well-defined and the margin is V-shaped. The free length of digit IV is greater than that of digit II. Depth of track depression is 17 mm proximal to the metatarsophalangeal pad and shallows to 14 mm towards the distal digits.

Type material: holotype BP/6/735 silicon mould housed at the Evolutionary Science Institute (University of Witwatersrand). Paratype: BP/6/735.

Type horizon and locality: upper Elliot Formation, Stormberg Group (Lower Jurassic). Trackway site (29° 27′ 08.57″S, 27° 42′ 08.51″E) is on an informal road between the villages of Ha Mokhosi and Ha Matobo (Maseru District, Lesotho; Fig 1). The site is immediately adjacent to the Matobo trackway site briefly documented by Ambrose [22].

Etymology: *ambro*—Ambrose (derived from the Latin name Ambrosius) meaning “immortal”; This is in honour of Emeritus Professor David Ambrose for his detailed recording of the localities of several of the Roma trackways. It was during an attempt at the relocation of these sites that the newly exposed trackway was discovered. And, *kholohali*, from the Sesotho ‘kholo’, meaning ‘big/large/great’; and ‘hali’, meaning “much/very” after their unexpectedly large size.

### Role of the metatarsophalangeal pad ratios in diagnoses

**Fig 7:**
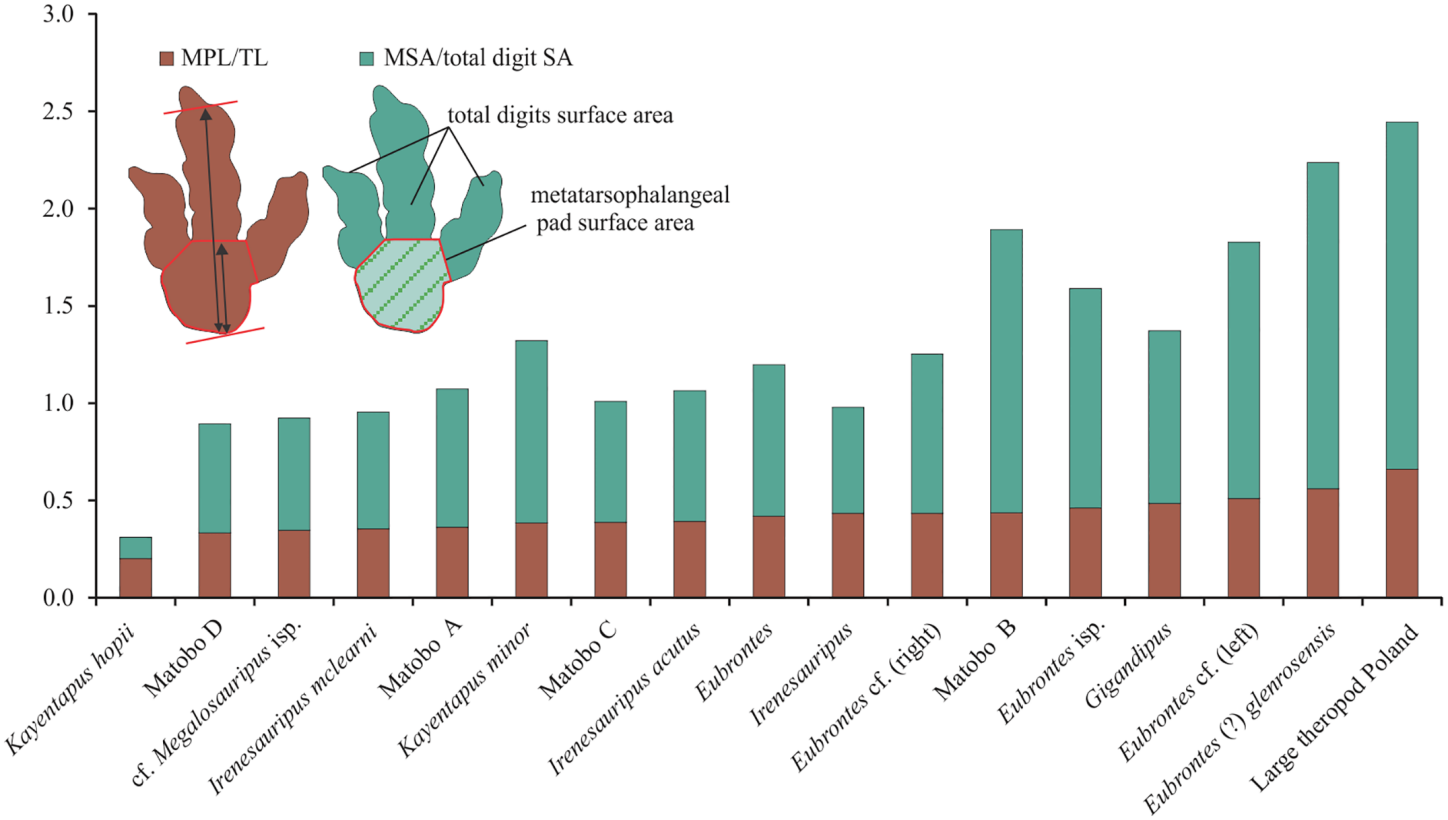
Metatarsophalangeal (MSA) to total digit surface and metatarsophalangeal pad length (MPL) to total length (TL) comparisons for Late Triassic-Early Jurassic and Lower Cretaceous ichnotaxa. Values were derived from parameters measured in photographs of publications listed in Table 3.

Because larger animals are more likely to have better developed metatarsophalangeal pads [76], we examine here the importance of the utility of measurements drawn from the metatarsophalangeal pad length (MPL) and metatarsophalangeal surface area (MSA) (Table 3; Fig 7). However, it is important to note that the heel and digit surface areas may be variable between tracks and sites, and that several ways of measuring dimensions can be made from line drawings. This is because these ratios, much like the track impression as a whole, are dependent on several external factors, i.e. animal weight and speed, sediment properties, sediment collapse around digits and secondary factors relating to their degree of preservation. Lallensack et al. [41] point out that the size of the heel surface area may vary due to the additional impression of the metatarsus or partial impression of the foot.

Morphologically, the metatarsophalangeal pad (MP) of several Jurassic and Cretaceous ichnotaxa show large surface area impressions but narrow length dimensions (Table 3; Fig 7). Large ichnites have MPL that can represent ≥33% of the track length, e.g., *Megalosauripus* [62] and *Eubrontes* (?) *glenrosensis* [66]. Whereas other, Lower Jurassic ichnotaxa have values of ≤29% (i.e., *Eubrontes*; [77]). Matobo A, B, and C have a very large MPL relative to the total TL with percentages varying from 36% (A), 44% (B) to 39% (C) (Table 3; Fig 7). Matobo D has 33% of total track length represented by the metatarsophalangeal pad (Table 3; Fig 7).

Generally, the greatest variability between ichnotaxa is shown in the ratio MSA to total digital surface areas (Fig 7), whereas the ratio of MPL:TL shows more consistency despite the differences reflected in surface areas. With respect to the ratio of the MPL:TL (Fig 7), and not taking age in consideration, *Kayentapus hopii* has the smallest metatarsophalangeal pad/ total foot length, whereas *Eubrontes* (?) *glenrosensis* and the Polish theropod track of Niedźwiedzki [61] have the largest. The MP to total digital surface area ratio for Matobo D falls within a ± 5% range with the following ichnotaxa: *Irenesauripus*, cf. *Megalosauripus* isp., *I*. *mclearni*, and *I*. *acutus*, confirming their moderately small metatarsophalangeal pad area/ total digit surface area. Similarities in these morphometrics are more marked between Matobo D and *Irenesauripus* isp., cf. *Megalosauripus* isp. and *Kayentapus* isp.

**Fig 8:**
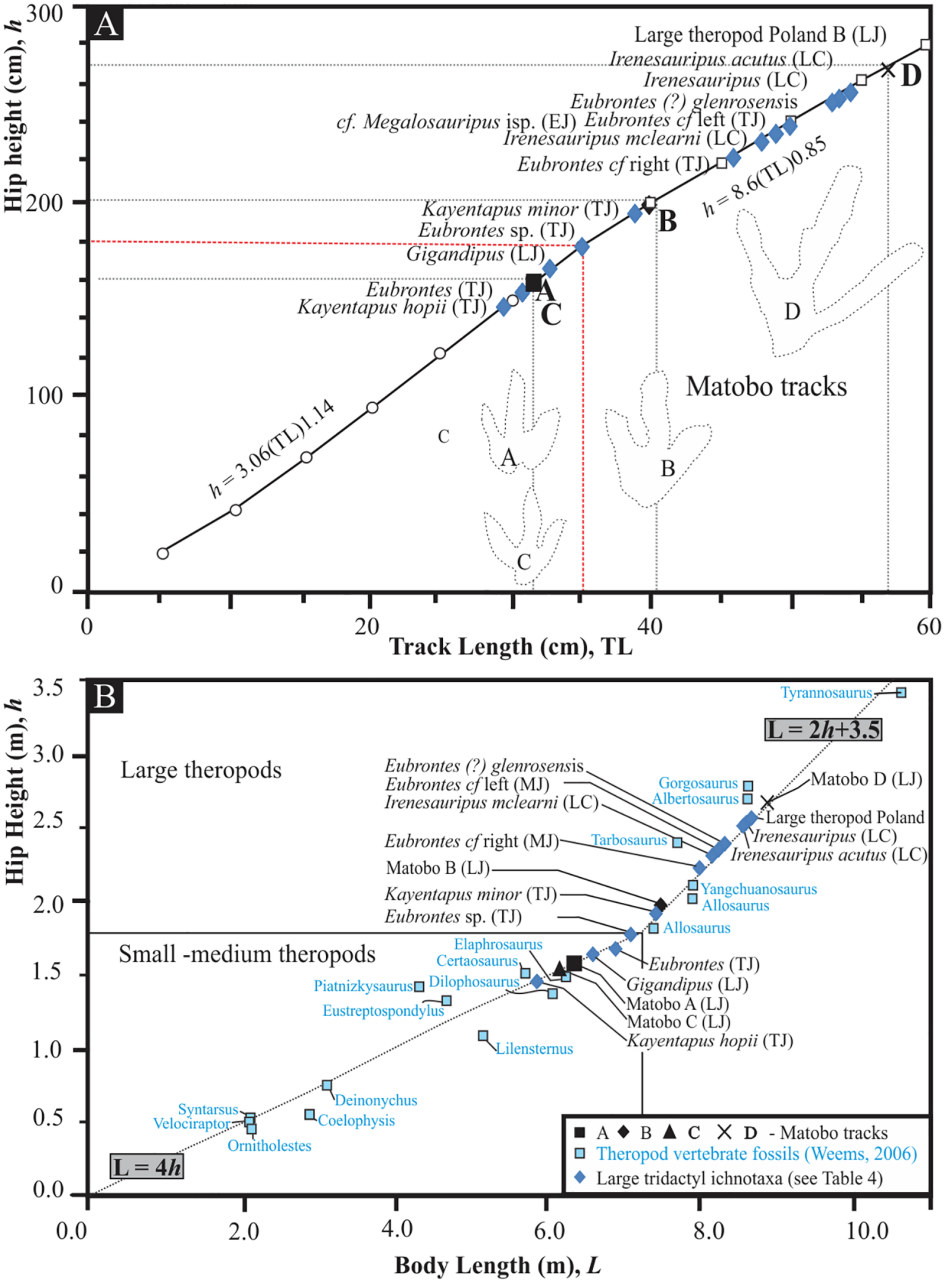
Comparisons of calculated hip height and body length of various ichnotaxa. (A) Estimated hip heights of trackmakers from the track lengths of the four trackways at Matobo (A–D) and other globally occurring large theropod tracks. (B) Estimated morphometric hip heights and body lengths of various Jurassic and Cretaceous theropod dinosaurs (light blue squares) taken from Weems [45] against calculated hip height and body length values from trackways of theropod dinosaurs reported in Table 4. Abbreviations: TJ—Triassic-Jurassic, LJ—Lower Jurassic; MJ—Middle Jurassic; LC—Lower Cretaceous. For calculations, refer to Table 4; for equations, see Methods. Scale bar = 15 cm.

Large metatarsophalangeal pad area to total digit surface area suggests a bulky, robust ‘heel’ pad, and when contrasted against a low length ratio can imply a ‘heel’ pad dimension which is short but wide (i.e. as in *Eubrontes* isp., *Eubrontes* (?) *glenrosensis* and the large Polish theropod track; Fig 7). Interestingly, the taxa most dissimilar to Matobo D are those which exhibit broad, thick digits of equal surface area to the metatarsophalangeal pad, or ones for which the MPL makes up a significant proportion of TL (Figs 7 and 8); e.g., *Eubrontes* cf., *Eubrontes* (?) *glenrosensis* and the large theropod taxa described by Niedźwiedzki [61] from Poland.

### Trackmaker identity

The Matobo tracksite represents some of the larger Early Jurassic theropod tracks in southern Africa. Certainly, Matobo D represents the largest Early Jurassic track, globally. Other globally occurring contemporaneous megatheropod tracks (cf. *Megalosauripus* isp. and large Polish theropod; Table 4) are solely represented by the Sołtyków site tracks, Holy Cross Mountains (Poland), which are 54 cm in size [60, 78]. These tracks were found in the Zagaje Formation which is considered to be Hettangian in age based on sequence stratigraphic analysis [79] and its early ‘Liassic’ (Early Hettangian) flora [80], which has been critiqued [81]. Other similar sized ichnites are represented by the much younger Lower Cretaceous material (e.g. [72, 82]). In addition to the Matobo tracks, the hip heights and body lengths of the theropod track makers have been estimated from several other globally occurring large theropod ichnites, and these had estimated hip heights (*h*) of 1.5–2 m and body lengths that ranged from ~6 to 9 m (Table 4; Fig 8A and 8B). When compared to hip heights and body lengths reconstructed from fossil material [45], the calculated heights and lengths obtained from track lengths show a linear trend (Fig 8B).

Reconstructed body dimensions for Matobo A and C give estimated hip heights similar to those estimated for the ichnogenera *Eubrontes* isp. (see [54]), *Kayentapus hopii* (from [67]) and *Gigandipus* ([68]; Tables 3 and 4; Fig 8A). Estimated hip heights for Matobo B are comparable with estimations from *K*. *minor* ([61]; Fig 8A; Tables 3 and 4). Comparable vertebrate fossil hip and body length for the track maker of Matobo B is the allosaurid *Allosaurus* (as is the case for *K*. *minor*, *Eubrontes*, and *Gigandipus*; Table 4). Matobo A and C are more likely to be associated with an animal of dimensions resembling *Dilophosaurus* (see discussion below), *Ceratosaurus* (Upper Jurassic; Morrison Formation, USA; Lourinhã and Alcobaça formations, Portugal) [83]; or *Elaphrosaurus* (Tendaguru Formation, Tanzania, [84]; Fig 8B).

Matobo D, being the largest of the Matobo tracks, yields a very large track maker with a hip height of up to 2.7 m and a body length of ~9 m (Fig 8A; Table 4). Other comparable theropod ichnites for which hip height and body length range could be extrapolated are the Polish large theropod, *Irenesauripus*, *I*. *mclearni*, *I*. *acutus*, *Eubrontes* cf. (left), *Eubrontes* (?) *glenrosensis* (Fig 8A; Table 4).

Remarkably, estimated body length and hip heights for Matobo D are comparable to (age dissociated) tyrannosaurid and allosaurid vertebrate fossil material, and represents the upper limit of the Upper Jurassic *Allosaurus* (occurring in North America, Portugal, and Tanzania; [85]). The unprecedentedly large dimensions of Matobo D are all the more noteworthy when contrasted against known theropod body fossils from the latest Triassic and earliest Jurassic. Well-preserved theropod material from this time period is rare, and there is a distinct lack of comparable vertebrate body fossil material from the Lower Jurassic. Gierliński et al. [62] have proposed that some large Lower Jurassic tracks may also be considered to be made by (basal) allosaurids, but there are little to no body fossil evidence to support this claim despite the occurrence of these large tracks. Globally, the large theropod dinosaur trackmakers appear more consistently in the Middle to Upper Jurassic, and are mainly thought to be allosaurids and megalosaurids with robust metatarsus and phalanges and inferred well-developed metatarsophalangeal pads [64, 76].

In general, the limited vertebrate fossil evidence for larger prints (≥30 cm TL) has meant that many have been, in part, attributed to the relatively large (6 m body length, ~400 kg; [86]) Early Jurassic theropod *Dilophosaurus*. On the basis of their foot anatomy, the tracks of these animals are considered likely to be robust, with a large metatarsophalangeal pad area/total digit surface area. It is not considered to produce gracile tracks, but rather larger, robust tracks such as Matobo B (Figs 7 and 8).

Allosauroid-like material from the Elliot Formation of South Africa has been tentatively reported by Ray and Chinsamy [16] based on SAM-PK-K10013, which is a large, recurved and finely serrated tooth. Ray and Chinsamy [16] suggest that the tooth possibly belongs to a basal theropod but its stratigraphic position within the Elliot Formation is unknown. Other large, serrated teeth have been found within the Elliot Formation (e.g. SAM 383; [87]), although their stratigraphic and biological affinities are unstudied/unknown, and it remains plausible that some of this material is crurotarsan (i.e., “rauisuchid”) in nature. In general, skeletal remains of carnivorous dinosaur fossils are rare in the Upper Triassic—Lower Jurassic of southern Africa, whereas ichnites of these animals are much more common. To date, the oldest tridactyl theropod ichnite reported from South Africa is from the Upper Triassic uppermost Molteno Formation (Maclear trackway site, TL between 16–19.5 cm; [19]), whereas the youngest theropod tracks are in the lower Clarens Formation of Lesotho and South Africa (e.g., [20, 24, 88]). None of these southern African tridactyl theropod ichnites are associated with theropod skeletal remains.

The first theropod body fossils in South Africa are found in the Lower Jurassic upper Elliot Formation (UEF) and are represented by only two theropod genera, *Dracovenator regenti* [18] and *Coelophysis* (*Syntarsus*) *rhodesiensis* [89]. *Coelophysis* is suspected as the maker of the abundant *Grallator* isp. [30] traces in the UEF, although this ichnite, due to the range of sizes, probably represents a variety of small and medium-sized theropods [20, 30, 89]. In contrast, *D*. *regenti* is known only from fragmentary skull material with an estimated total skull length of 500 mm [18]. Yates [18] considers the general dimensions of *Dracovenator* similar to the theropod *Dilophosaurus wetherilli* (5–6 m in length) and, indeed, *Dracovenator* has been recovered from within the putatively monophyletic “dilophosaurid” clade [86]. The track lengths estimated for *Dracovenator*, in the size range of ~25–34 cm, would be similar to the TL reported for Matobo A, B (*Eubrontes*-like) and C (*Kayentapus*-like) here. The foot morphology of *Dilophosaurus* has also been suggested to share several morphological analogies with the ichnite *Kayentapus* [90]. Taken together, the morphological characteristics of Matobo D (in particular the size, narrowness and length of the digits), suggest that the tracks were made by a relatively gracile, carnivorous dinosaur with an allosaurid-like bauplan. Nonetheless, this suggestion awaits substantiation via the discovery of additional fossil theropod material from the poorly sampled basal rocks of the Jurassic.

## Conclusions

Our discovery of a new megatheropod trackway and several large tracks suggests that, in comparison to the Upper Triassic, the size range of the theropod trackways and, by extension that of their body size, rapidly expanded in the Early Jurassic. In southern Africa, where theropod body fossils are extremely rare, it also suggests an unappreciated degree of diversity of theropods active during this time. Currently, it is unclear whether the appearance of megatheropods is a consequence of a) “ecological release” following the extinction of non-crocodylomorph crurotarsans during the end-Triassic biocrisis event [5]; or b) a similar set of ecological stimuli that led to the progressive size increase of Sauropodomorpha beginning in the mid-Norian. In any case, our results suggest that very large theropods appeared in the fossil record of southern Africa prior to the Pliensbachian, mostly likely in a relatively short window of evolutionary time following the TJB, and not during the Middle Jurassic as currently suggested by the body fossil record. The appearance of mega-carnivores in the Early Jurassic is of great interest, and augurs an evolutionary phenomenon that was repeated on multiple occasions throughout the remainder of the Mesozoic, producing such iconic taxa as *Allosaurus* in the Upper Jurassic as well as *Spinosaurus* and *Tyrannosaurus* in the Upper Cretaceous. Furthermore, this study corroborates recent assessments of Elliot Formation biostratigraphy, with the semi-arid environment of the upper Elliot Formation able to support both small and very large sized theropods, as well as a diverse array of early sauropodomorphs (i.e. McPhee et al. [40]). Ecological incentives to increase body size may thus be related to rapid changes in both climate and predator-prey dynamics at the outset of the Jurassic, with the expansion of dinosaurian niche space accommodating a range of novel strategies not possible prior to the TJB [91]. This also has bearing on the manner in which sauropodomorph size thresholds represent a good predictor of theropod size increases and vice versa, with this relationship warranting in-depth future investigation.

## References

[1] Sereno PC. Basal archosaurs: phylogenetic relationships and functional implications. Journal of Vertebrate Paleontology. 1991 12 31;11(S4):1–53.

[2] Brusatte SL, Benton MJ, Ruta M, Lloyd GT. Superiority, competition, and opportunism in the evolutionary radiation of dinosaurs. Science. 2008 9 12;321(5895):1485–8. doi: 10.1126/science.1161833 18787166 10.1126/science.1161833

[3] Brusatte SL, Benton MJ, Ruta M, Lloyd GT. The first 50 Myr of dinosaur evolution: macroevolutionary pattern and morphological disparity. Biology Letters. 2008 12 23;4(6):733–6. doi: 10.1098/rsbl.2008.0441 18812311 10.1098/rsbl.2008.0441 PMC2614175

[4] Lloyd GT, Davis KE, Pisani D, Tarver JE, Ruta M, Sakamoto M, et al Dinosaurs and the Cretaceous terrestrial revolution. Proceedings of the Royal Society of London B: Biological Sciences. 2008 11 7;275(1650):2483–90. 10.1098/rspb.2008.0715 PMC2603200 18647715

[5] Olsen PE, Kent DV, Sues HD, Koeberl C, Huber H, Montanari A, et al Ascent of dinosaurs linked to an iridium anomaly at the Triassic-Jurassic boundary. Science. 2002 5 17;296(5571):1305–7. doi: 10.1126/science.1065522 12016313 10.1126/science.1065522

[6] Benton MJ. Origin and relationships of Dinosauria. The Dinosauria. 2004;2:7–19.

[7] Charig AJ. Competition between therapsids and archosaurs during the Triassic period: a review and synthesis of current theories. In: Symposia of the Zoological Society of London 1984 (No. 52, pp. 597–628). Cambridge University Press.

[8] Lucas SG, Klein HE, Lockley MG, Spielmann JA, Gierlinski GD, Hunt AP, et al Triassic-Jurassic stratigraphic distribution of the theropod footprint ichnogenus Eubrontes. New Mexico Museum of Natural History and Science Bulletin. 2006;37:86–93.

[9] Lucas SG, Tanner LH. The nonmarine Triassic—Jurassic boundary in the Newark Supergroup of eastern North America. Earth-Science Reviews. 2007 9 30;84(1):1–20.

[10] Benson RB, Campione NE, Carrano MT, Mannion PD, Sullivan C, Upchurch P, et al Rates of dinosaur body mass evolution indicate 170 million years of sustained ecological innovation on the avian stem lineage. PLoS Biol. 2014 5 6;12(5):e1001853 doi: 10.1371/journal.pbio.1001853 24802911 10.1371/journal.pbio.1001853 PMC4011683

[11] Schwartz HL, Gillette DD. Geology and taphonomy of the *Coelophysis* quarry, Upper Triassic Chinle Formation, Ghost Ranch, New Mexico. Journal of Paleontology. 1994 9 1;68(05):1118–30.

[12] Raath MA. Morphological variation in small theropods and its meaning in systematics: evidence from *Syntarsus*. Carpenter K, Currie PJ, editors. Cambridge: Cambridge University Press; 1990.

[13] Sciscio L, Bordy EM, Reid M, Abrahams M. Sedimentology and ichnology of the Mafube dinosaur track site (Lower Jurassic, eastern Free State, South Africa): a report on footprint preservation and palaeoenvironment. PeerJ. 2016 8 23;4:e2285 doi: 10.7717/peerj.2285 27635310 10.7717/peerj.2285 PMC5012264

[14] Lockley MG. New perspectives on morphological variation in tridactyl footprints: clues to widespread convergence in developmental dynamics. Geological Quarterly. 2010 3 27;53(4):415–32.

[15] Raath MA. The anatomy of the Triassic theropod Syntarsus rhodesiensis (Saurischia: Podokesauridae) and a consideration of its biology. 1977. (Doctoral dissertation, Rhodes University). pp. 1–233.

[16] Ray S, Chinsamy A. A theropod tooth from the Late Triassic of southern Africa. Journal of Biosciences. 2002 6 1;27(3):295–8. 12089478 10.1007/BF02704918

[17] Bristowe A, Raath MA. A juvenile coelophysoid skull from the Early Jurassic of Zimbabwe, and the synonymy of *Coelophysis* and *Syntarsus*. Palaeontologia africana. 2004; 40, 31–41.

[18] Yates AM. A new theropod dinosaur from the Early Jurassic of South Africa and its implications for the early evolution of theropods. Palaeontologia africana. 2005; 41: 105–122.

[19] Raath MA, Kitching JW, Shone RW, Rossouw GJ. Dinosaur tracks in Triassic Molteno sediments: the earliest evidence of dinosaurs in South Africa? Palaeontologia africana. 1990; 27: 89–95.

[20] Ellenberger P. Les niveaux paléontologiques de première apparition des mammifères primordiaux en Afrique du Sud et leur ichnologie: établissement de zones stratigraphiques détaillées dans le Stormberg du Lesotho (Afrique du Sud)(Trias supérieur à Jurassique). InIUGS, 2nd symposium on gondwana stratigraphy and palaeontology 1970 (pp. 343–370). Pretoria: Council for Scientific and Industrial Research.

[21] Wilson JA, Marsicano CA, Smith RM. Dynamic locomotor capabilities revealed by early dinosaur trackmakers from Southern Africa. PLoS ONE. 2009 10 6;4(10):e7331 doi: 10.1371/journal.pone.0007331 19806213 10.1371/journal.pone.0007331 PMC2752196

[22] Ambrose, D. A note on fossil trackways at Roma. Lesotho Miscellaneous Documents No. 4, House 9 Publications Roma, 2003 iv+ 14pp.

[23] Duncan RA, Hooper PR, Rehacek J, Marsh J, Duncan AR. The timing and duration of the Karoo igneous event, southern Gondwana. Journal of Geophysical Research: Solid Earth 1997;102: 18127–18138

[24] Ellenberger, P. Contribution à la classification des Pistes de Vértebrés du Trias: les Stormberg d'Afrique du Sud (I). Paleovertebrata, Memoire Extraordinaire 1972, Montpellier. 1972 Pp. 152.

[25] Ellenberger P. Contribution à la classification des pistes de vertébrés du Trias: les types du Stormberg d'Afrique du Sud. Le stormberg superieur-I. Le bioma de la zona B/1 ou niveau de Moyeni: ses biocénesis. 2ème Partie. Laboratoire de paléontologie des vertébrés; Palaeovertebrata, Memoire ExtraordinaireMontpellier. 1974 pp. 170.

[26] Smith RM, Marsicano CA, Wilson JA. Sedimentology and paleoecology of a diverse Early Jurassic tetrapod tracksite in Lesotho, southern Africa. Palaios. 2009 10; 24(10):672–84.

[27] Marsicano CA, Wilson JA, Smith RM. A temnospondyl trackway from the early mesozoic of western Gondwana and its implications for basal tetrapod locomotion. PloS ONE. 2014 8 6;9(8):e103255 doi: 10.1371/journal.pone.0103255 25099971 10.1371/journal.pone.0103255 PMC4123899

[28] Abrahams M, Bordy EM, Sciscio L, Knoll F. Scampering, trotting, walking tridactyl bipedal dinosaurs in southern Africa: ichnological account of a Lower Jurassic palaeosurface (upper Elliot Formation, Roma Valley) in Lesotho. Historical Biology. 2017 1 4:1–8.

[29] Botha BJ. The provenance and depositional environment of the Red Beds stage of the Karroo System. In Int. Geol. Congr., Argentina, Symp. on Gondwana strat. and palaeont 1967; 763–774. Palaeontologia Africana. 1984; 25: 87–110.

[30] Olsen PE, Galton PM. A review of the reptile and amphibian assemblages from the Stormberg of southern Africa, with special emphasis on the footprints and the age of the Stormberg. Palaeontologia Africana. 1984; 25, 87–110.

[31] Kitching JW, Raath MA. Fossils from the Elliot and Clarens Formations (Karoo Sequence) of the northeastern Cape, Orange Free State and Lesotho, and a suggested biozonation based on tetrapods. Palaeontologia africana. 1984; 25, 111–125.

[32] Smith R, Kitching J. Sedimentology and vertebrate taphonomy of the Tritylodon acme zone: a reworked palaeosol in the Lower Jurassic Elliot Formation, Karoo Supergroup, South Africa. Palaeogeography, Palaeoclimatology, Palaeoecology. 1997 6 1;131(1–2):29–50.

[33] Lucas SG, Hancox PJ. Tetrapod-based correlation of the nonmarine Upper Triassic of southern Africa. Albertiana. 2001; 25:5–9.

[34] Knoll F. Review of the tetrapod fauna of the “Lower Stormberg Group” of the main Karoo Basin (southern Africa): implication for the age of the Lower Elliot Formation. Bulletin de la Societe géologique de France. 2004 1 1;175(1):73–83.

[35] Knoll F. The tetrapod fauna of the Upper Elliot and Clarens formations in the main Karoo Basin (South Africa and Lesotho). Bulletin de la Société géologique de France. 2005 1 1;176(1):81–91.

[36] Bordy EM, Hancox PJ, Rubidge BS. Fluvial style variations in the Late Triassic—Early Jurassic Elliot Formation, main Karoo Basin, South Africa. Journal of African Earth Sciences. 2004 3 31;38(4):383–400.

[37] Bordy EM, Hancox PJ, Rubidge BS. A description of the sedimentology and palaeontology of the Late Triassic—Early Jurassic Elliot Formation in Lesotho. Palaeontologia africana, 2004: 40; 43–58.

[38] D'Orazi Porchetti SD, Nicosia U. Re-examination of some large early Mesozoic tetrapod footprints from the African collection of Paul Ellenberger. Ichnos. 2007 5 30;14(3–4):219–45.

[39] Bordy EM, Eriksson P. Lithostratigraphy of the Elliot Formation (Karoo Supergroup), South Africa. South African Journal of Geology. 2015 9 1;118(3):311–6.

[40] McPhee BW, Bordy EM, Sciscio L, Choiniere JN. The sauropodomorph (Dinosauria) biostratigraphy of the Elliot Formation of southern Africa: tracking the evolution of Sauropodomorpha across the Triassic—Jurassic boundary. Acta Palaeontologica Polonica. 2017;62(3):441–65.

[41] Lallensack JN, van Heteren AH, Wings O. Geometric morphometric analysis of intratrackway variability: a case study on theropod and ornithopod dinosaur trackways from Münchehagen (Lower Cretaceous, Germany). PeerJ. 2016 6 8;4:e2059 doi: 10.7717/peerj.2059 27330855 10.7717/peerj.2059 PMC4906676

[42] Mallison H, Wings O. Photogrammetry in paleontology—a practical guide. Journal of Paleontological Techniques. 2014; 12:1–31.

[43] Thulborn RA. Preferred gaits of bipedal dinosaurs. Alcheringa. 1984 1 1;8(3):243–52.

[44] Thulborn T. Dinosaur tracks. 1990 Chapman and Hall, London (p. 410).

[45] Weems RE. The manus print of *Kayentapus minor*; its bearing on the biomechanics and ichnotaxonomy of Early Mesozoic saurischian dinosaurs. The Triassic—Jurassic Terrestrial Transition. New Mexico Museum of Natural History and Science Bulletin. 2006;37:369–78.

[46] Thulborn RA, Wade M. Dinosaur trackways in the Winton Formation (mid-Cretaceous) of Queensland. Memoirs of the Queensland Museum. 1984;21(2):413–517.

[47] Alexander R. Estimates of speeds of dinosaurs. Nature. 1976 5;261:129–30.

[48] Bordy EM, Sciscio L, Abdala F, McPhee BW, Choiniere JN. First Lower Jurassic vertebrate burrow from southern Africa (upper Elliot Formation, Karoo Basin, South Africa). Palaeogeography, Palaeoclimatology, Palaeoecology. 2017 2 15;468:362–72.

[49] Belvedere M, Farlow J. A numerical scale for quantifying the quality of preservation of vertebrate tracks. Dinosaur Tracks: The Next Steps. 2016 8 15:93.

[50] Weems R. E. A re-evaluation of the taxonomy of Newark Supergroup saurischian dinosaur tracks, using extensive statistical data from a recently exposed tracksite near Culpeper, Virginia. 1992.

[51] Wagensommer A, Latiano M, Mocke HB, D'Orazi P. Dinosaur diversity in an Early Jurassic African desert: the significance of the Etjo Sandstone ichnofauna at the Otjihaenamaparero locality (Namibia). Neues Jahrbuch für Geologie und Paläontologie-Abhandlungen. 2016 8 1;281(2):155–82.

[52] Piubelli D, Avanzini M, Mietto P. The Early Jurassic ichnogenus *Kayentapus* at Lavini di Marco ichnosite (NE Italy); global distribution and palaeogeographic implications. Bollettino della Società geologica italiana. 2005 1 1;124(1):259–67.

[53] Lockley MG, Cart K, Martin JA, Milner AR. New theropod tracksites from the Upper Cretaceous “Mesaverde” Group, western Colorado: implications for ornithomimosaur track morphology. Fossil Record 3. New Mexico Museum of Natural History and Science, Bulletin. 2011;53:321–9.

[54] Olsen PE, Smith JB, McDonald NG. Type material of the type species of the classic theropod footprint genera *Eubrontes*, *Anchisauripus*, and *Grallator* (Early Jurassic, Hartford and Deerfield basins, Connecticut and Massachusetts, USA). Journal of Vertebrate Paleontology. 1998 9 15;18(3):586–601.

[55] Moratalla JJ, Sanz JL, Jimenez S. Multivariate analysis on Lower Cretaceous dinosaur footprints: discrimination between ornithopods and theropods. Geobios. 1988 1 1;21(4):395–408.

[56] Sternberg CM. Dinosaur tracks from Peace River, British Columbia. In: Annual Report of the National Museum of Canada 1932; 59–85.

[57] Wagensommer A, Latiano M, Leroux G, Cassano G, D’orazi Porchetti SI. New dinosaur tracksites from the Middle Jurassic of Madagascar: Ichnotaxonomical, behavioural and palaeoenvironmental implications. Palaeontology. 2012 1 1;55(1):109–26.

[58] Wagensommer A, Latiano M, Mocke HB, Porchetti SD, Wanke A. A Dinosaur Ichnocoenosis from the Waterberg Plateau (Etjo Formation, Lower Jurassic), Namibia. Ichnos. 2016 10 1;23(3–4):312–21.

[59] Farlow JO, Gatesy SM, Holtz TR Jr, Hutchinson JR, Robinson JM Theropod locomotion 1. American Zoologist. 2000 8;40(4):640–63.

[60] Gierlinski G, Niedzwiedzki G, Pienkowski G. Gigantic footprint of a theropod dinosaur in the Early Jurassic of Poland. Acta Palaeontologica Polonica. 2001;46(3).

[61] Niedźwiedzki G. Ślady wielkich teropodów z wczesnojurajskich osadów Gór Świętokrzyskich. Przegląd Geologiczny. 2006;54(7):615–21.

[62] Gierliński G, Pieńkowski G, Niedźwiedzki G. Tetrapod track assemblage in the Hettangian of Sołtyków, Poland, and its paleoenvironmental background. Ichnos. 2004 7 1;11(3–4):195–213.

[63] Lockley MG, Meyer CA, dos Santos VF. *Megalosauripus*, *Megalosauropus* and the concept of megalosaur footprints. In The Continental Jurassic: Symposium Volume: Museum of Northern Arizona Bulletin 1996; 60: 113–118.

[64] Lockley MG, Meyer CA, Dos Santos VF. *Megalosauripus* and the problematic concept of megalosaur footprints. Gaia. 1998;15:313–37.

[65] Turner S, Bean LB, Dettmann M, McKellar JL, McLoughlin S, Thulborn T. Australian Jurassic sedimentary and fossil successions: current work and future prospects for marine and non-marine correlation. GFF. 2009 6 1;131(1–2):49–70.

[66] Adams TL, Strganac C, Polcyn MJ, Jacobs LL. High resolution three-dimensional laser-scanning of the type specimen of Eubrontes (?) glenrosensis Shuler, 1935, from the Comanchean (Lower Cretaceous) of Texas: implications for digital archiving and preservation. Palaeontologia Electronica. 2010 1 1;13(3):11.

[67] Lockley M.G., Gierlinski G.D., and Lucas S.G. *Kayentapus* revisited: notes on the type material and the importance of this theropod footprint ichnogenus. In: Sullivan R.M., Lucas S.G., and Spielmann J.A. (eds.), Fossil Record 3. New Mexico Museum of Natural History and Science Bulletin. 2011; 53: 330–336.

[68] Milner AR, Harris JD, Lockley MG, Kirkland JI, Matthews NA. Bird-like anatomy, posture, and behavior revealed by an Early Jurassic theropod dinosaur resting trace. PloS ONE. 2009 3 4;4(3):e4591 doi: 10.1371/journal.pone.0004591 19259260 10.1371/journal.pone.0004591 PMC2645690

[69] Rainforth EC. Ichnotaxonomy of the fossil footprints of the Connecticut Valley (Early Jurassic, Newark Supergroup, Connecticut and Massachusetts). Ph.D. thesis, Columbia University. 2005.

[70] Lockley MG, Gierlinski GD, Dubicka ZO, Breithaupt BH, Matthews NA. A preliminary report on a new dinosaur tracksite in the Cedar Mountain Formation (Cretaceous) of eastern Utah. New Mexico Museum of Natural History and Science, Bulletin. 2014 11 1;62:279–85.

[71] McCrea RT, Buckley LG, Farlow JO, Lockley MG, Currie PJ, Matthews NA, et al A ‘terror of tyrannosaurs’: the first trackways of tyrannosaurids and evidence of gregariousness and pathology in Tyrannosauridae. PLoS ONE. 2014 7 23;9(7):e103613 doi: 10.1371/journal.pone.0103613 25054328 10.1371/journal.pone.0103613 PMC4108409

[72] Razzolini NL, Oms O, Castanera D, Vila B, dos Santos VF, Galobart À. Ichnological evidence of Megalosaurid Dinosaurs Crossing Middle Jurassic Tidal Flats. Scientific reports. 2016;6: 31494 doi: 10.1038/srep31494 . 27538759 10.1038/srep31494 PMC4990902

[73] Niedzwiedzki G, Remin Z. Gigantic theropod dinosaur foot prints from the upper Pliensbachian of the Holy Cross Mountains, Poland. Prz. Geol. 2008;56(9):823–5.

[74] Lockley MG, Gierlinski GD, Houck KA, Lim JD, Kim KS, Kim DY, et al New excavations at the Mill Canyon Dinosaur Track site (Cedar Mountain Formation, Lower Cretaceous) of Eastern Utah. New Mexico Museum of Natural History and Science, Bulletin. 2014 11 1;62:287–300

[75] McCrea RT. Vertebrate palaeoichnology of the Lower Cretaceous (lower Albian) Gates Formation of Alberta (Doctoral dissertation). 2000.

[76] Xing L, Zhang J, Lockley MG, McCrea RT, Klein H, Alcalá L, et al Hints of the early Jehol Biota: important dinosaur footprint assemblages from the Jurassic-Cretaceous Boundary Tuchengzi Formation in Beijing, China. PloS ONE. 2015 4 22;10(4):e0122715 doi: 10.1371/journal.pone.0122715 25901363 10.1371/journal.pone.0122715 PMC4406591

[77] Lockley MG, Mickelson D. Dinosaur and pterosaur tracks in the Summerville and Bluff (Jurassic) beds of eastern Utah and northeastern Arizona. Mesozoic geology and paleontology of the Four Corners region. 1997;48:133–8.

[78] Niedźwiedzki G. Ślady wielkich teropodów z wczesnojurajskich osadów Gór Świętokrzyskich. Przegląd Geologiczny. 2006;54(7):615–21.

[79] Gierliński G, Pieńkowski G. Dinosaur track assemblages from the Hettangian of Poland. Geological Quarterly. 1999 2 14;43(3):329–46.

[80] Wcislo-Luraniec E. Flora from Odrowaz in Poland-a typical Lower Liassic European flora. Proceedings of the Pan-European Palaeobotanical Conference, Vienna. September 1991: 331–335.

[81] Pacyna G. Critical review of research on the Lower Jurassic flora of Poland. Acta Palaeobotanica. 2013 12 1;53(2):141–63.

[82] Cobos A, Lockley MG, Gascó F, Royo—Torres R, Alcalá L. Megatheropods as apex predators in the typically Jurassic ecosystems of the Villar del Arzobispo Formation (Iberian Range, Spain). Palaeogeography, Palaeoclimatology, Palaeoecology. 2014 4 1;399:31–41.

[83] Mateus O. Late Jurassic dinosaurs from the Morrison Formation (USA), the Lourinha and Alcobaça formations (Portugal), and the Tendaguru Beds (Tanzania): a comparison. New Mexico Museum of Natural History and Science Bulletin. 2006;36:223–31.

[84] Rauhut OW. Theropod dinosaurs from the Late Jurassic of Tendaguru (Tanzania). Special Papers in Palaeontology. 2011 1 1;86:195–239.

[85] Hendrickx C, Hartman SA, Mateus O. An overview of non-avian theropod discoveries and classification. PalArch’s Journal of Vertebrate Palaeontology. 2015 1 1;12(1):1–73.

[86] Brusatte SL, Nesbitt SJ, Irmis RB, Butler RJ, Benton MJ, Norell MA. The origin and early radiation of dinosaurs. Earth-Science Reviews. 2010 7 31;101(1):68–100.

[87] Nesbitt SJ, Brusatte SL, Desojo JB, Liparini A, De França MA, Weinbaum JC, et al Rauisuchia. Geological Society, London, Special Publications. 2013 1 1;379(1):241–74.

[88] Raath MA, Yates AM. Preliminary report of a large theropod dinosaur trackway in Clarens Formation sandstone (Early Jurassic) in the Paul Roux district, northeastern Free State, South Africa. Palaeontologia Africana. 2005; 41: 101–104.

[89] Munyikwa D, Raath MA. Further material of the ceratosaurian dinosaur Syntarsus from the Elliot Formation (Early Jurassic) of South Africa. Palaeontologia africana. 1999; 35: 55–59.

[90] Gierliński G, Niedźwiedzki G, Pieńkowski G. Early Hettangian vertebrate ichnoassemblage from Poland. In Conference of Tracking dinosaur origins. The Triassic/Jurassic terrestrial transition 2005; Mar 14: pp. 3–4.

[91] Sheridan JA, Bickford D. Shrinking body size as an ecological response to climate change. Nature Climate Change. 2011 11 1;1(8):401–406.

